# Development and validation of the Multidimensional Gender Inequality Perception Scale (MuGIPS)

**DOI:** 10.1371/journal.pone.0301755

**Published:** 2024-04-18

**Authors:** Sofía Schwartz-Salazar, Efraín García-Sánchez, Rocío Martínez, Rosa Rodríguez-Bailón

**Affiliations:** 1 Department of Social Psychology, University of Granada, Granada, Spain; 2 Research Center of Mind, Brain, and Behavior (CIMCYC), University of Granada, Granada, Spain; Murcia University, Spain, SPAIN

## Abstract

Perceptions of gender inequality may motivate people to take action against inequality given its negative impact on various domains of people’s everyday lives. Thus, it is crucial to develop reliable measures that consider the multidimensional nature of gender inequalities. In this research, we propose and assess the psychometric properties of the Multidimensional Gender Inequality Perception Scale (MuGIPS). This is a self-reported measure of perceived gender inequality in four domains: health, violence, household work and caregiving, and public sphere and power. Exploratory and confirmatory factor analyses were conducted to test the validity and reliability of the MuGIPS with Spanish participants in three samples (N = 1733). The analyses revealed that the MuGIPS had a good internal consistency and showed four factors associated with gender inequality in the four specified domains. Moreover, scores in all the dimensions positively correlated with feminist identity and feminist ideology, as much as with some attitudinal variables. In contrast, results showed a negative correlation with system-justifying ideologies. The MuGIPS shows adequate reliability and validity for measuring the perception of gender inequality in the health, violence, household work and caregiving, and public sphere and power domains among Spanish university and general population samples.

## Introduction

Gender inequalities have diminished in recent decades alongside the rise of feminist and social movements [1, 2]. Simultaneously, awareness of gender inequalities and aggressions faced by women in their daily lives has grown, exemplified by global feminist mobilizations such as “Me Too” and the Spanish movement “*Hermana*, *yo sí te creo*” (Sister, I believe you) [3, 4]. Nevertheless, gender-based inequalities, which especially affect women, persist across various domains of everyday life. International institutions and associations emphasize the imperative of reducing current inequalities and preventing the reversal of the progress made to date [1, 5]. Importantly, one factor that could hold back gender equality is people’s misperceptions of inequalities between men and women. Perceptions of inequality have been proven to be one of the main factors that motivate individuals to reduce inequality, especially economic inequality [6]. To assess similar processes regarding gender inequality, researchers need to count on reliable perceived gender inequality instruments that account for the multidimensionality of gender inequality. However, to our knowledge, there are only a few scales that focus on measuring the perception of gender inequality, without measuring at the same time other constructs, across different domains of everyday life. In addition, there are no measures of gender inequality that include the dimensions used in the most recent indexes of objective gender inequality indicators. Therefore, our research aimed to fill this gap by proposing and evaluating the psychometric properties of a new instrument, the Multidimensional Gender Inequality Perception Scale (MuGIPS), and subsequently test whether this perception is related to other variables of interest for gender inequality reduction. This instrument assesses the extent to which respondents believe gender differences exist between men and women across various domains of everyday life (health, violence, household work and caregiving, education, paid job and economics, and power and representation) within a specific society.

Gender inequality stems from stereotyped social and cultural roles and refers to the unequal treatment or opportunities given to individuals based on their gender, primarily disadvantaging women, and manifesting in various forms [7]. Thus, gender inequality is a multidimensional phenomenon that can affect various domains of everyday life. The multidimensionality of gender inequality is reflected in indicators such as the Gender Inequality Index (with three domains: reproductive health, empowerment, and the labour market [8]) and the Gender Equality Index [5], which assesses gender inequality levels in 6 core domains (work, money, knowledge, time, power, and health) and 2 additional domains (violence and intersecting inequalities).

In addition to the structural inequalities captured by indicators, perceptions of inequality can be extremely important in predicting people’s responses to inequality [6, 9–12], as well as other psychological variables related to subjective wellbeing [13, 14]. However, the literature on perceptions of inequality has mostly focused on economic disparities, often overlooking the importance of gender inequalities as a multidimensional phenomenon. Nonetheless, we believe that understanding the implications and consequences of gender inequalities across multiple domains, such as health, caregiving and household tasks, work and public life participation, and gender violence, is crucial.

Furthermore, gender-based health inequalities are noteworthy. While life expectancy for women tends to be higher compared to men, it does not necessarily indicate better overall health [15]. Medical textbooks are gender-biased [16], and women are underrepresented in the study of diseases and drug trials [17]. Additionally, there is a tendency to misdiagnose women for certain diseases usually attributed to men (see Yentl Syndrome [18]) and a lack of studies and diagnosis of diseases that specifically affect women [19]. Moreover, violence against women is another significant dimension of gender inequality, with studies highlighting higher rates of workplace harassment [20] and intimate partner violence against women compared to men [21]. In the household work and caregiving domain, women traditionally assume more caregiving responsibilities, leading to increased barriers in balancing personal and professional realms, as well as impacting leisure time and health [5, 22]. Also, it is relevant to highlight gender inequalities in domains closely tied to the traditionally male-associated public sphere, including education, paid jobs and economics, and power and representation [23]. Despite increased female enrolment in education, horizontal segregation persists in the career choices for men and women (e.g., STEM), as well as vertical segregation concerning the positions they hold within companies, organisations, and institutions [8, 24]. This inequality is also linked to the gender pay gap [25] and the underrepresentation of women in politics [26].

Regardless of the importance of multidimensionality in understanding inequality, existing measures for gender inequality perception often overlook its diverse manifestations across various domains. For instance, some instruments only consider gender inequality in a single or just a few domains, such as income gaps or occupational differences [27, 28]. Moreover, some measures consist of general quantitative questions about perceived gender inequality (e.g., “Do you think that, in general, men and women are treated equally in your country?” [29]) or employ single indicators combining different dimensions and using only a “yes/no” response format (e.g., “Do men and women enjoy equal status in their family and society?” [30]). Other instruments tend to mix perceived gender inequality with other variables focus on respondents’ personal experiences, beliefs and sexist ideologies; or account for different dimensions that do not correspond to the dimensions used in objective indexes’ indicators [31], for example, leaving aside the health dimension. Our research overcomes these limitations by providing a measure with appropriate psychometric properties that address the multidimensionality of gender inequality perceptions. In this regard, first, the MuGIPS offers a multidimensional perspective, covering classic domains such as income, socio-political representation, and unpaid caregiving work inequalities; while adding relevant domains like health, violence, and education. These dimensions align with the included in most of the recent indexes of objective gender inequality indicators. Second, we provide a valid measure for the Spanish context in which there are no similar instruments. Third, we examine the relationship between gender inequality perceptions and other relevant variables such as political attitudes and individual differences. This instrument could be used to assess how much gender inequality people perceive, and especially how much they underestimate it as much as it correlates with other variables specifically related to inequality reduction and wellbeing.

To summarize, in the current research, we aimed to (a) create a comprehensive and multidimensional instrument to measure the perceived gender inequality across various everyday life domains (i.e., health, violence, household work and caregiving, paid job and economics, education, and power and representation) and (b) test the instrument’s psychometric properties (i.e. exploring and confirming the measure’s structure, assessing the measurement invariance between men and women, and collecting evidence of the scale’s reliability and validity in the Spanish context), which implied examining the relationships between perceived gender inequality and demographics, ideological, identity, and attitudinal variables.

## Methods

### Participants

We conducted three studies with different samples comprised of Spanish participants (N_total_ = 1,733) aged 18 to 73 years (*M*_age_ = 27.57; *SD*_age_ = 10.71), of which 59.6% were women, and 65.89% university students (see Table 1 for subsamples’ demographics; additional demographics in S1 Table).

**Table 1:** Main demographics by sample.

	Sample 1	Sample 2	Sample 3
	(N = 673)	(N = 498)	(N = 558)
Purpose	Exploratory Factor Analysis	Confirmatory Factor Analysis	Discriminant Validity
Sample type	University Students	General Population	University students (84.1%) and Staff (16%)
Gender	57.8% women	53.2% women	67.9% women; 1.4% other; 1.8% I prefer not to answer
Age (*M*, *SD*)	23.29 (5.39)	35.35 (13.23)	24.9 (9.04)
	min = 18; max = 63	min = 18; max = 71	min = 18; max = 73

### Instruments

The measures included in this research are the following:

#### Multidimensional Gender Inequality Perception Scale (MuGIPS)

This measure, developed for the present research and used across the three samples, evaluates perceived gender inequality. Inspired by the Gender Equality Index by the EIGE [5], the MuGIPS was designed to cover six domains in which gender inequalities are observed (health, violence, household work and caregiving, paid job and economics, education, and power and representation). Participants indicated how often several situations of inequality occur in the context of their country. The MuGIPS has a 7-point Likert response format ranging from 1 (*never*) to 7 (*always*).

To develop this instrument, we defined the construct of perceived gender inequality as "being aware of the existence of differences between women and men in terms of roles, opportunities, and outcomes in different domains of life (health, violence, household work and caregiving, education, paid job and economics, and power and representation) within a given society" and generated an item pool. Subsequently, we submitted our definition and items to evaluation by 12 experts (66.6% women) in social psychology, inequality, gender issues and psychometrics. These experts evaluated the construct and each item’s (a) representativeness of the construct, (b) representativeness of the inequality domain, (c) degree of belonging to the selected domain, (d) understanding and (e) clarity, all using a 4-points Likert answer scale from 1 (*do not agree at all*) to 4 (*totally agree*). Considering the experts’ advice, we modified and unpacked some items and re-conceptualized some dimensions. As a result, we theoretically organized the resulting items in the mentioned six domains (health, violence, household work and caregiving, paid job and economics, education, and power and representation). Following Polit et al. [32] recommendations, we selected 30 items with a kappa value higher than .70.

Preliminary descriptive and logistic regression analyses (see S1 File) of the MuGIPS were conducted with Sample 1 to identify whether sociodemographic variables were associated with the likelihood of not responding to the items and reduce potential item bias. In this first study, we added a response option, “I prefer not to answer”. We also had a comments section where participants could indicate whether the items were understood, easy to answer, or if they had any other observations, which we used as an indication of the correct functioning of the items. We analyzed item response frequencies (see S2 Table), and combined with descriptive data we used them to detect response patterns and floor and ceiling effects. The mean scores for all items were above 2 and under 6, except for Items 6 and 8, which belong to the Violence dimension. We decided to keep these items for their conceptual, practical, and contextual relevance, given that they are related to sexual and street violence against women, two key aspects of gender inequality and gender violence highlighted in Spanish society [33]. Before conducting exploratory factorial analysis (EFA), we excluded two items because of the high response rate (> 5%) to the answer option “I prefer not to answer”(deleted items 1 and 2), one item because of its low correlation with the rest of the items on the scale (*r* < .300; deleted item 4), and two more items following the experts’ qualitative criteria–one of them was evaluated as too general (deleted item 5) and the content of a second one seemed to correspond to another item already included in the final selection (deleted item 3). The subsequent EFA, conducted with the 25 final items and Sample 1’s responses, revealed a four-factor structure; with the dimensions of health (*r* = .74, *p* < .001), violence (α = .90), and household work and caregiving (α = .82) well represented. The fourth factor, which was reconceptualized and renamed as “public sphere and power” (α = .95), consisted of a conglomerate of the education, economics and paid job, and public sphere and power dimensions. McDonald’s Omega for the scorings in altogether items was appropriate ω_t_ = .97 (α = .97). To detect possible strange behaviours of the items that could affect the validity of the scores obtained, a study of the differential functioning of the items (DIF) in relation to gender and income was performed. We tested the 25 items that were finally selected using Sample 3 data. Using the Lordif package for R software [34] we observed the McFadden pseudo R2 in all items was below .02 allowing us to classify the DIF as negligible according to regular standards (< .13) [35]. The MuGIPS items are presented in Table 2 (for the original version in Spanish see S3 Table). Unless otherwise noted, all variables were calculated as mean scores of the items.

**Table 2:** Final version of the MuGIPS’ items.

Dimension	Item wording	#
Health	Less attention is paid to the same health problem in women than in men.	1
	Women’s specific health problems are underestimated compared to men’s specific health problems	2
Violence	Women experience more insecurity because of the possibility of being assaulted in everyday contexts (in the street, at work. . .) than men.	6
	Women suffer more from violence than men because they are women	7
	Women are sexually assaulted to a greater extent than men.	8
	Women face more workplace harassment than men.	9
	Women are treated as objects to a greater extent than men.	10
	Women suffer more violence than men in intimate partner relationships.	11
Household work and caregiving	Women take more responsibility for the caregiving of their children than men.	16
	Women do more housework than men.	17
	Women are more likely than men to engage in caregiving for family members and others close to them.	18
Public sphere and power	(PR) The work-life balance is more difficult for women than for men.	19*
	(PR) Women are under more pressure than men to give up their careers to take care of their families.	20*
	(PR) Men have greater representation and power than women in private and public institutions.	21
	(PR) Although they have the same rights, men are socially respected more than women.	22
	(PR) Women are questioned more than men when they do not do what is expected of them.	23
	(PR) Men’s opinions and ideas are more valued than those of women.	24
	(PR) In general, men have more power than women in our society.	25
	(Edu) Girls and boys are educated differently about their roles in society.	3
	(Edu) University studies that are mainly taken by men are more valued than those taken by women.	4
	(Edu) Women encounter more obstacles than men in pursuing their studies.	5
	(EL) Women face more barriers to finding employment than men.	12
	(EL)Women face more obstacles than men in accessing the most socially valued jobs.	13
	(EL) In our society there is a gender pay gap, i.e., men are paid more than women even though they do the same work.	14
	(EL) Men are more likely than women to have access to a type of employment with better working conditions.	15

*Note*: PR = theorized Power and Representation domain; Edu = theorized Education domain; EL = theorized Economics and Labor domain; * Items written to capture intersectional gender inequalities between the public sphere and power dimension, and household work and caregiving dimension.

#### Sexism

Sexism was measured with the Spanish version of the Ambivalent Sexism Inventory (ASI [36]), comprising 22 items grouped into two subscales: hostile sexism, which acknowledges the view that conceives women as a threat to men and upholds the superiority of men over women (e.g., *Women try to gain power by controlling men*), and benevolent sexism, which refers to women as fragile, precious, and morally superior compared to men (e.g., *Women should be loved and protected by men*; α = .92; Sample 3).

#### Feminist ideology

The feminist ideology was measured through the Knowledge and Attitudes Toward Feminism scale (IMCAF [37]), an instrument developed in the Spanish context that accounts for the beliefs, knowledge, and attitudes that respondents have towards equality between men and women and feminism as a socio-political movement and ideology. This scale includes 17 items grouped in two subscales: knowledge about feminism (e.g., *"Feminism" is the opposite of "machismo"*) and attitudes toward feminism (e.g., *To me*, *feminism is something negative*; α = .91; Sample 3).

#### Feminist identification

We used a translated short version of the Multicomponent Ingroup Identification Scale [38] to measure feminist identification. As done in various research concerning the Spanish population [39], we used three items from the solidarity subscale (e.g., *I feel solidarity with feminists*), three items from the centrality subscale (e.g., *The fact that I am a feminist is an important part of my identity*), and an extra general item (i.e., *I identify with feminists*) to assess to what extent participants feel they are similar to feminists and how important it is to be a feminist for them (α = .96; Sample 3).

#### Social dominance orientation

The social dominance orientation variable was assessed with a translated version [40] of the short SDO7 Scale by Ho et al. [41]. It is composed of 8 items that measure the endorsement of domination, acceptance of hierarchies, and preference for inequality between groups (e.g., *Some groups of people are simply inferior to other groups*; α = .75; Sample 3).

#### Meritocratic beliefs

The meritocratic beliefs were measured with an instrument developed by Lalonde et al. [42] translated into Spanish. Participants indicated to what extent they agree with 6 different meritocratic affirmations about the belief that individual talent and effort determine the outcomes (e.g., *Effort is key to success*; α = .91; Sample 3).

#### Beliefs in a Just World

The Belief in a Just World is a measure by Dalbert [43], that has 6 items in total measuring whether participants think people get what they deserve (e.g., *In general*, *people get what they deserve*; α = .81; Sample 3).

#### Attitudes towards affirmative actions

The attitudes towards affirmative action variable was measured by using the adapted version by Moya and Expósito [44] of the Attitudes toward Affirmative Action scale [45], which includes 3 items about initiatives that promote the inclusion and wellbeing of women in the workplace and economics (e.g., *Are you in favour of equal opportunity programs for women*; α = .92; Sample 3).

#### Collective action to reduce gender inequality

The collective action to reduce gender inequality was measured with an 8-item adaptation from the scale used by Jiménez-Moya [46]. Participants were asked: *To what extent would you be willing to participate in the following action*? The scale includes items such as *Sign an online petition to reduce gender inequality*, with higher scoring indicating greater support for mobilization to confront inequalities between men and women (*α* = .93; Sample 3).

The response format for all these instruments is a 7-point Likert scale, in which participants indicate the extent of their agreement with each item. This scale ranges from 1 (*strongly disagree*) to 7 (*totally agree*). For all instruments, a mean score was calculated.

#### Threat to men’s collective interest

To assess the perceived threat to men’s collective interest derived from affirmative action measures we used Moya and Expósito’ [44] version of the Men’s Collective Interest scale [45]. The scale consists of 6 items divided into 3 affirmations (e.g., *Affirmative action programs disadvantage men*, *compared to women*, *in terms of their chances of getting a job*) and 3 questions (e.g., *To what extent are you convinced that the implementation of these programs gives women greater opportunities for promotion and advancement*?). For affirmations, the response ranges from 1 (*totally disagree*) to 7 (*totally agree*). For question-items, the response scale goes from 1 (*not convinced at all*) to 7 (*totally convinced*). After reverse scoring, an index with the sum of the 6 items (affirmations plus questions) was calculated. Higher scores indicate a higher sense of threat to men’s collective interest (α = .67; Sample 3).

#### Sociodemographics

Finally, across the three samples, participants provided information about their gender, age, nationality, mother tongue, sexual orientation, political orientation (ranging from 1 = *far-left* to 7 = *far-right*), occupation, educational level (ranging from 1 = *none* to 9 = *PhD*), income, number of family members, and subjective socioeconomic status [47] (ranging from 1 = *lowest socioeconomic status position* to 10 = *highest socioeconomic status position*).

### Procedure

Participants were reached through university e-mail services (Samples 1, 2 and 3) and social media networks (including Twitter, Instagram, WhatsApp, and Facebook; Samples 1 and 2). Samples 1 and 2 were collected concurrently between 30 November 2022 and 31 January 2023. This data was split into a group of university students (Sample 1) and a group of non-students. Sample 3 was collected from 7 to 31 March 2023. Participation in the study was voluntary and questionnaires were completed online. Participants signed an online informed consent before taking the survey and were prompted to answer the questions as honestly as possible given that there were no correct or incorrect answers. Participants entered a drawing for a 50-euro prize for responding to the survey. At the end of the questionnaire, they were debriefed and thanked for their collaboration. No identifying information was collected. These studies were approved by the Ethical Committee of the University of Granada (Approval N° 969/CEIH/2023) following the Declaration of Helsinki and were preregistered (https://osf.io/xrds6/?view_only=644f1a013ba34bc0bc9150c1fe26f92d).

### Data analysis

To examine the structure of the MuGIPS, we conducted analyses on R version 4.0.4. and R Studio [48], with psych, lavaan, and sjPlot R packages [49–51].

Additionally, we performed correlation and regression analyses to test the discriminant validity of the instrument. The items for each measure across Samples were highly consistent, indicating enough reliability according to Cronbach’s alpha and McDonald’s Omega. We conducted an exploratory factor analysis (EFA) to examine the reliability and underlying dimensionality of the scale (Sample 1). Confirmatory factor analysis (CFA) was used to test the robustness of the factorial structure (Sample 2), and regression analyses were conducted to evaluate the relationships between the scale and sociodemographic and attitudinal variables, as well as model invariance. Finally, we tested the MuGIPS’ discriminant validity by conducting a correlational analysis and with the Heterotrait-Monotrait Ratio of Correlations (HTMT [52]; Sample 3).

The collected data, codebook and supporting information are available at https://osf.io/xrds6/?view_only=644f1a013ba34bc0bc9150c1fe26f92d

## Results

### Descriptive statistics

Table 3 displays the descriptive statistics of the items and reliability scores for Samples 1 and 2. The skewness values were generally acceptable (between -2 and +2 [53]) for all items, except for item 8 in Sample 1 (skew = - 2.20). This, together with the kurtosis of the same item in Samples 1 and 2, suggests a strong consensus regarding women being sexually assaulted to a greater extent than men in Spanish society. Similarly, an elevated kurtosis is observed for item 11 (“Women suffer more violence than men in intimate partner relationships”) in Sample 1.

**Table 3:** Items’ descriptive statistics in Sample 1 (EFA) and Sample 2 (CFA).

	*Sample 1*					*Sample 2*				
*Item*	*M (SD)*	*CITC*	*alpha*	*skew*	*kurtosis*	*M(SD)*	*CITC*	*alpha*	*skew*	*kurtosis*
1	2.94 (1.67)	.64	.96	0.64	-.74	2.75 (1.67)	.647	.96	0.93	-0.11
2	3.55 (1.87)	.709	.96	0.28	-1.13	3.30 (1.82)	.715	.96	0.50	-0.89
3	4.80 (1.81)	.672	.96	-0.59	-.74	4.54 (1.70)	.699	.96	-0.47	-0.65
4	4.22 (1.86)	.737	.96	-0.25	-1.10	3.96 (1.77)	.736	.96	-0.08	-0.98
5	3.75 (1.63)	.715	.96	0.05	-0.91	3.50 (1.65)	.702	.96	0.27	-0.87
6	6.25 (1.13)	.653	.96	-1.72	2.17	5.90 (1.35)	.667	.96	-1.30	1.20
7	5.77 (1.55)	.766	.96	-1.52	1.77	5.53 (1.55)	.754	.96	-1.31	1.29
8	6.41 (0.91)	.667	.96	-2.20	6.65	6.33 (0.95)	.645	.96	-1.84	4.27
9	5.79 (1.31)	.705	.96	-1.23	1.38	5.49 (1.36)	.737	.96	-0.98	0.80
10	5.53 (1.42)	.765	.96	-1.10	0.97	5.26 (1.53)	.758	.96	-0.93	0.32
11	5.89 (1.15)	.676	.96	-1.74	4.23	5.65 (1.26)	.726	.96	-1.28	1.88
12	4.86 (1.53)	.819	.96	-0.62	-.13	4.62 (1.50)	.783	.96	-0.50	-0.30
13	5.18 (1.52)	.838	.96	-0.89	0.25	4.94 (1.61)	.872	.96	-0.70	-0.24
14	4.88 (1.60)	.75	.96	-0.81	0.09	4.59 (1.72)	.757	.96	-0.56	-0.61
15	4.67 (1.44)	.797	.96	-0.70	0.00	4.57 (1.50)	.801	.96	-0.57	-0.30
16	5.46 (1.19)	.641	.96	-1.09	1.43	5.46 (1.19)	.683	.96	-1.19	1.90
17	5.61 (1.09)	.66	.96	-1.10	1.99	5.60 (1.17)	.696	.96	-1.17	1.88
18	5.70 (1.08)	.651	.96	-1.19	1.79	5.71 (1.08)	.683	.96	-1.19	1.85
19	5.28 (1.55)	.727	.96	-0.87	0.11	5.33 (1.56)	.684	.96	-0.94	0.29
20	5.04 (1.55)	.699	.96	-0.65	-0.30	5.04 (1.51)	.751	.96	-0.74	0.04
21	5.51 (1.42)	.77	.96	-1.16	1.08	5.39 (1.42)	.769	.96	-1.04	0.74
22	5.02 (1.66)	.817	.96	-0.80	-0.17	4.63 (1.66)	.772	.96	-0.60	-0.48
23	5.23 (1.69)	.793	.96	-0.90	-0.06	4.91 (1.73)	.75	.96	-0.75	-0.44
24	4.59 (1.60)	.831	.96	-0.55	-0.42	4.44 (1.57)	.808	.96	-0.49	-0.48
25	5.41 (1.52)	.824	.96	-1.14	0.78	5.36 (1.40)	.805	.96	-1.03	0.81

*Note*: M = mean scoring; SD = standard deviation; CITC = Corrected Item Total Correlation; alpha = Cronbach’s alpha when the item is deleted.

### Exploratory factor analysis (EFA)

The data showed suitable properties to perform EFA (KMO = .97; Bartlett’s test, χ^2^_(300)_ = 12865.45 p < .001). Parallel analysis suggested a four-factor structure explaining a total of 64% of the variance across dimensions (health 9%, violence 14%, household work and caregiving 10%, and public sphere and power 30%).

**Fig 1:**
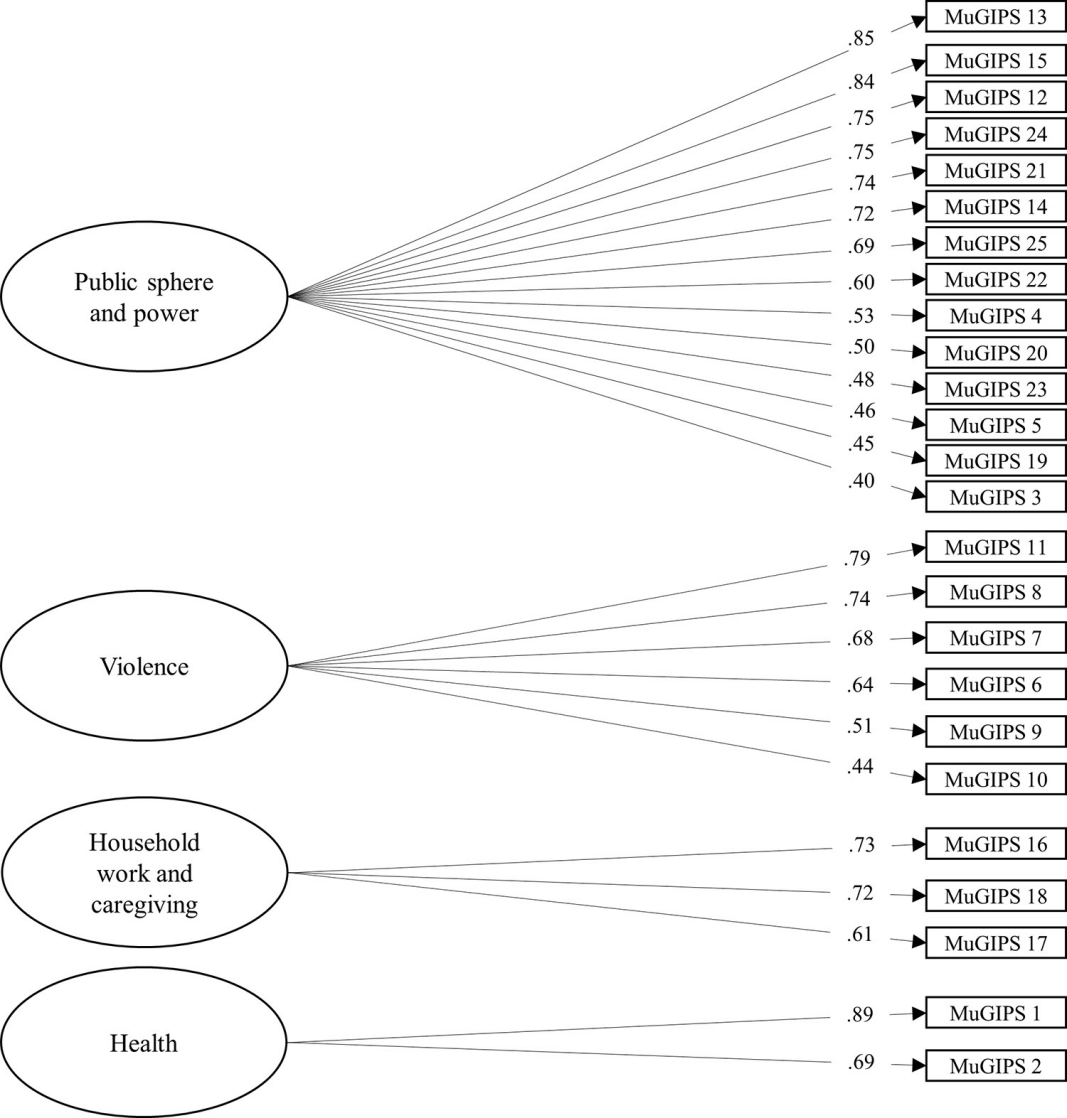
Factor loadings and communality for Sample 1 (EFA).

A first-order EFA was conducted with Sample 1 (N = 673) using the final selection of 25 items. According to the Mardia test (Skewness = 8503.06, *p* < 0.001; Kurtosis = 58.12, *p* < 0.001), our data showed a non-normal distribution. To handle non-normality [54], we employed a robust maximum likelihood method with robust standard errors. Factor loadings for the tested items are shown in Fig 1. With the exception of item 19, saturation was equal to or higher than .40 for all items, exclusively in their respective dimensions, when using an oblimin rotation. Item 19, which addresses intersecting domains of time, domestic and labour, showed low factor loadings in the public sphere and power dimension and the household work and caregiving dimension. This could be explained by the item’s intersectional nature. Considering that the item had a greater factor load in the public sphere and power dimension, we included it in this factor.

In sample 1, the items were consistently related and provided appropriate reliability for scores in MuGIPS (α = .97; ωt = .97) according to regular standards (i.e. α > = .7, and ωt > = .7) [55, 56]. For the health dimension, consisting of two items, a correlation index was calculated (*r* = .74, *p* < .001). The violence (α = .90; ω = .90), household work and caregiving (α = .82; ω = .82), and public sphere and power dimensions (α = .95; ω = .95) demonstrated a good internal consistency. The reliability indexes for scores in each item were higher than α = .95 (see Table 3). Overall, these indexes indicate acceptable reliability.

### Confirmatory factorial analysis (CFA)

The model derived from the EFA was assessed through CFA in Sample 2. A four-factor model, consisting of 25 items, was fitted using the maximum likelihood estimation with robust standard errors, not assuming a multivariate normality distribution given the Mardia test results (Skewness = 6742.44, *p* < 0.001; Kurtosis = 39.76, *p* < 0.001). As shown in Table 3, data regarding skewness and kurtosis for each item in Sample 2 did not present serious normality deviations.

To test the model’s consistency and confirm the MuGIPS’ structure, we considered the following fit indexes [57]: Comparative Fit Index (CFI), Tucker-Lewis Index (TLI), Error of Root Mean Square Approximation (RMSEA), and Standardized Root Mean Square Residuals (SRMR). The four-factor model presents an adequate fit across all tested indices (χ^2^_(224)_ = 621.064, p < .001, CFI = .953, TLI = .946, RMSEA (90% CI) = .060 (.053,—.067), SRMR = .038, AIC = 29472.2, BIC = 29684.4); and showed better adjustment than the single-factor model and second-order factor model (see S4 Table), confirming the structure proposed by the EFA.

### Measurement invariance

**Fig 2:**
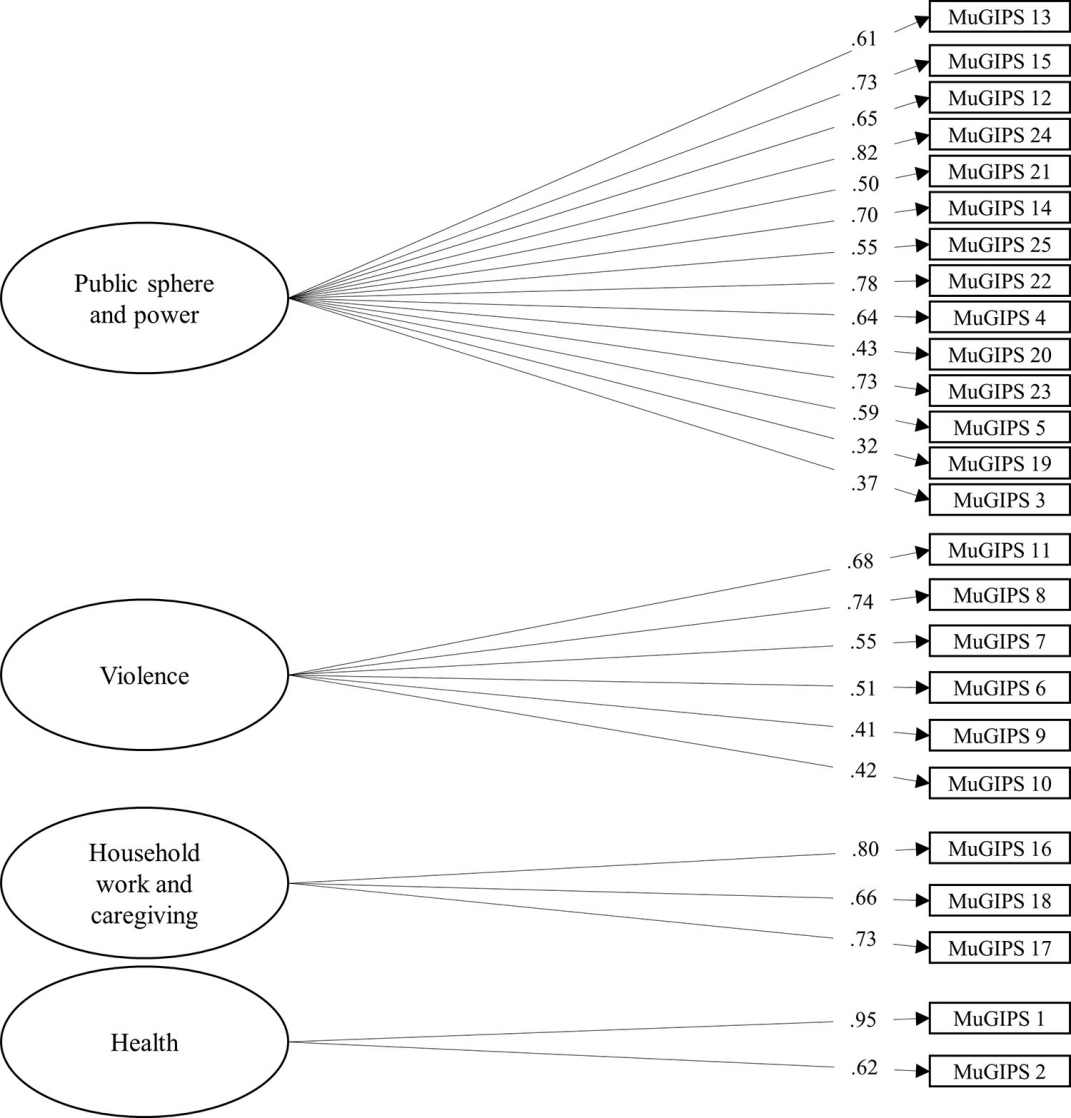
Factor loadings and communality for Sample 2 (CFA).

To assess the invariance of our model, we looked at adjustment indices for different models, using the four-factor model confirmed with Sample 2 as the base model (see Fig 2). Subsequently, we tested the adjustment for men and women separately, followed by configural and metric invariance tests. As recommended by Cheung and Rensvold [58], none of the four models tested and compared to the base model had a change greater than .01 in CFI. This implies that the model’s structure and loading pattern are consistent for both men and women, with each item contributing similarly to the latent construct in both groups (see Table 4). We did not find scalar invariance between men and women, possibly due to the nature of the measured construct, in which women are expected to score differently because of their experienced gender-based inequality and discrimination.

**Table 4:** Invariance model tested.

Model	χ^2^	df	*p*	CFI	TLI	RMSEA	SRMR
Base model	760.425	269	< .001	.949	.943	.061	.037
Model 2 (men)	573.613	269	< .001	.931	.923	.071	.048
Model 3 (women)	530.432	269	< .001	.951	.945	.058	.043
Model 4 (configural invariance)	1.104.045	538	< .001	.942	.935	.064	.043
Model 5 (metric invariance)	1.138.794	559	< .001	.94	.936	.064	.055
Model 6 (scalar invariance)	1214.12	580	< .001	.933	.931	.066	.058

### Correlates of the MuGIPS

We employed a Multiple Indicators of Multiple Causes (MIMIC) model, using age, gender, political orientation, income, subjective socioeconomic status, and educational level as predictors of the four MuGIPS factors. We found that women and left-wing supporters had a higher perception of gender inequality regarding health (B_gender_ = .57, *p* < .001, CI95% [.316, .820]; B_political orientation_ = -.39; *p* < .001; CI95% [-.469, -.318]), public sphere and power (B_gender_ = .65; *p* < .001; CI95% [.446, .864]; B_political orientation_ = -.34; *p* < .001; CI95% [-.417, -.263]), and household work and caregiving (B_gender_ = .49; *p* < .001; CI95% [.304, .680]; B_political orientation_ = -.23; *p* < .001; CI95% [-.306, -.152]). Lastly, scores in the violence dimension were higher for younger people (B_age_ = -.01; *p* = .003; CI95% [-.019, -.004]), women (B_gender_ = .34; *p* < .001; CI95% [.159, .514]), people leaning toward left-wing political orientation (B_political orientation_ = -.28; *p* < .001; CI95% [-.345, -.210]) and those with higher income (B_income_ = .04; *p* = .025; CI95% [.004, .067]).

### Relationship with other variables and discriminant validity

To obtain further evidence of the MuGIPS’s validity, we conducted a series of bivariate correlations between the total scale score and the subfactors scores with various ideological, identity, attitudinal, and demographic variables. As shown in Table 5, all dimensions of the MuGIPS positively correlated with each other and the total score. Additionally, these dimensions and total scoring negatively correlated to ambivalent sexism, beliefs in a just world (BJW), social dominance orientation (SDO), meritocratic beliefs, and threat to men’s collective interest by affirmative action. Moreover, all dimensions of perceived gender inequality negatively correlated with participants’ political orientation, indicating that left-wing voters tended to perceive more gender inequality. However, the MuGIPS and each of its dimensions positively correlated with feminist ideology and feminist identity, support for collective action to reduce gender inequality, attitudes towards affirmative action, subjective socioeconomic status, and gender. Regarding age, it negatively correlated only with the violence dimension,–i.e., younger participants perceive higher gender inequality regarding the violence men and women experience. Respondents’ income negatively correlated with all perceived gender inequality dimensions except for the health domain. Finally, the educational level did not correlate significantly with any of the MuGIPS scores. We calculated the mean differences in the MuGIPS by the categorical demographics (gender, sexual orientation, educational level and family annual income; See S2 File). We found that women perceive gender inequality to a greater extent than men. Bisexual people also perceive more gender inequality than heterosexual people. Lastly, people with lower incomes are more sensitized than people with somewhat higher annual incomes.

**Table 5:** Bivariate correlations among MuGIPS’ dimensions and other measured variables.

	MuGIPS total score	Health	Violence	Household Work and Caregiving	Public Sphere and Power	M (SD)
**MuGIPS total score**						5.24 (1.20)
**Health**	.686***					3.49 (1.73)
**Violence**	.851***	.432***				5.99 (1.13)
**Household Work and Caregiving**	.787***	.422***	.612***			5.66 (1.13)
**Public Sphere and Power**	.978***	.671***	.762***	.727***		4.95 (1.39)
**Ambivalent sexism**	-.579***	-.337***	-.540***	-.413***	-.565***	1.80 (0.76)
**Feminist identity**	.691***	.466***	.637***	.466***	.675***	5.35 (1.79)
**Feminist ideology**	.666***	.344***	.662***	.505***	.631***	5.94 (1.02)
**BJW**	-.251***	-.234***	-.210***	-.184***	-.245***	2.64 (0.81)
**SDO**	-.486***	-.317***	-.496***	-.329***	-.454***	2.24 (1.05)
**Meritocratic beliefs**	-.440***	-.417***	-.312***	-.304***	-.460***	3.01 (1.07)
**Support for collective action to confront gender inequality**	.727***	.478***	.670***	.489***	.708***	5.28 (1.58)
**Attitudes toward affirmative action**	.701***	.363***	.715***	.489***	.667***	5.75 (1.42)
**Threat to Men’s Interests**	-.278***	-.148***	-.246***	-.207***	-.277***	20.65 (5.73)
**SSS**	.147***	.107*	.112**	.108*	.150***	5.34 (1.39)
**Political orientation**	-.581***	-.409***	-.503***	-.405***	-.580***	3.92 (1.80)
**Age**	-.055	-.049	-.109*	.029	-.047	24.94 (9.03)
**Educational level**	.017	.031	.006	.027	.011	^*a*^
**Family annual income**	-.104*	-.082	-.083*	-.087*	-.099*	^*a*^
**Family members**	-.012	-.018	-.016	-.023	-.009	3.49 (1.28)

Note

*p>.05

**p>.01

***p>.005

^a^ ordinal variables

Given the high correlations found between the MuGIPS and the rest of the measured variables, we implemented an additional discriminant validity test by using the Heterotrait-Monotrait Ratio of Correlations (HTMT) [52]. All HTMT indexes were lower than .85, the threshold suggested in the literature [59–61] to assume that gender inequality perception as measured by the MuGIPS was different from ambivalent sexism (HTMT = .48), feminist ideology (HTMT = .69), feminist identity (HTMT = .71), support for collective action to confront gender inequality (HTMT = .76), attitudes towards affirmative action (HTMT = .74) and threat to men’s collective interest (HTMT = .75). This is, perceived gender inequality measured by the MuGIPS differs from other similar constructs already validated in the psychosocial literature.

## Discussion

Research on inequality perception has primarily focused on perceived economic inequality, showing the relevance of the perceptions of inequality [6] in relation to support for its reduction [9] as well as ideological [62, 63] and wellbeing variables [13, 14]. However, research specifically addressing the perception of gender inequality itself is sparser. Existing studies are approached either from a qualitative or a quantitative perspective but are limited to the study of only some of the domains in which inequality occurs [27–29] or use a dichotomous response scale [30]. Moreover, some studies examine the perception of gender inequality but intermingled with other constructs such as sexism [31]. As a result, the findings obtained from such studies cannot be solely attributed to the perception of gender inequality. Additionally, they often do not include the various domains in which inequality manifests, or the results are not easily comparable either among participants or to the dimensions included in the objective indicators.

The main aim of the present research was to address a gap in the literature by introducing the Multidimensional Gender Inequality Perception Scale (MuGIPS), a novel instrument with robust psychometric properties for both women and men, which assesses gender inequality perception in the Spanish context. The MuGIPS comprises 25 items that evaluate various domains in which gender inequality is observed including health, violence, household work and caregiving, and public sphere and power (education, paid job and economics, and power and representation). These domains correspond to the dimensions established in some of the most complete objective indicators of gender inequality available to date [5].

The MuGIPS demonstrated strong evidence of content validity, internal validity, and validity based on its relationships with other variables. Content validity was assessed through an expert panel evaluation of the construct and the items, whereas internal validity was confirmed through EFA and CFA, following the classical test theory [64]. The analyses confirmed the internal structure of the scale, which consists of four factors. Three factors (health, violence, and household work and caregiving) have been theorized since the beginning of the instrument construction. The fourth factor emerged as a conglomerate of the theorized dimensions of education, paid jobs and economics, and power and representation. This conglomerate was reconceptualized and renamed the “public sphere and power” domain, corresponding to the historical division between the public and private spheres that feminism has long criticised [65].

The four factors identified through the EFA and confirmed in the CFA largely account for the variance in the participants’ responses. Additionally, the scale showed good fit indices and adequate internal consistency. Regarding the equivalence for both women and men, our data demonstrated metric and configural invariance. The lack of scalar invariance could be attributed to the nature of the construct, as gender inequality tends to affect women more significantly and harshly. Thus, as expected and confirmed by our data, women perceive more gender inequality, possibly due to their increased exposure to and impact from it [15], or because of women’s higher empathy and prosocial behaviour under certain conditions [66–68]. In this regard, the MIMIC model indicates that women and left-wing political leanings perceive more gender inequality across domains, and younger people perceive more gender inequality in the violence domain specifically. Age differences could account for the ability of the wide variety of recent awareness-raising campaigns, interventions, and laws [69–71] developed against gender violence to resonate with young people. However, it is still important to include other areas of gender inequality in these policies and interventions, which are also, in general, less known and less perceived. For its part, correlations between political orientation and feminism have been extensively documented. Even though feminist beliefs are not necessarily an intrinsic belief of left-wing movements, nor are feminist orientations only necessarily based on left-wing political orientation (see neoliberal feminism [72]; feminist identification and other feminism-related variables are frequently strongly associated with left-wing voters and more liberal political orientations (vs. conservative) [73] as our results also demonstrate.

We further enhanced the MuGIPS’s validation through its relationships with various validated instruments of diverse natures (ideological, identity, attitudinal, and sociodemographic). Notably, there was a positive relationship between the MuGIPS and feminist identification and ideology, as well as support for collective action to confront gender inequality and affirmative action measures. These results, together with the negative relationship between MuGIPS scores and system-justifying ideologies (meritocratic beliefs, SDO, and BJW) and age, show a strong consistency between perceived gender inequality levels and participants’ belief systems. These findings highlight the potential significance of perceived gender inequality in reducing gender inequality, akin to findings in studies on perceived economic inequality [62]. Moreover, it empirically reinforces the idea of perceived gender inequality as a characteristic closely related to feminism [74], highlighting the idea of feminism and gender inequality awareness as streams of social change (vs. system-justifying ideologies) and challenges to the status quo [75].

In summary, our manuscript contributes to the existing knowledge on perceptions of gender inequality by addressing previous gaps in the literature in at least three key ways. First, it provides a new instrument designed to assess perceived gender inequality: the MuGIPS. The scale focuses on gender inequality in four domains (health, violence, household work and caregiving, and power and public sphere), advancing shortcomings detected in previous scales that mix perceived gender inequality with other constructs or do not account for the dimensions affected by gender inequality. Thus, MuGIPS can help researchers to assess perceptions of gender inequality more accurately from a broader perspective. Second, the scale’s psychometric properties were robust across samples including, men and women, university students, and the general population. Thus, the scale is reliable for measuring gender inequality perceptions across different social groups. Additionally, to our knowledge, the MuGIPS is the first instrument with these characteristics to be fully developed in Spanish and following the open science principles. Third, we demonstrate that gender inequality perception differs from other relevant variables concerning gender issues, such as feminist ideology and identification or support for collective action. In this line, the MuGIPS could expand the knowledge regarding gender inequality perception by providing a reliable measure to assess gender inequality awareness and be useful for assessing the effectiveness of interventions promoting gender equality.

As a limitation, it is important to note that although factorially and in terms of reliability, the scale seems to behave adequately, there is a certain imbalance between the representation of each domain within the instrument. For example, the health dimension finally had only two items, which could compromise to some extent the reliability of this dimension. Moreover, future studies may use a test-retest approach to strengthen the reliability and reproducibility of the scores, as much as Item Response Theory to delve deeper item discrimination and generalizability. Nevertheless, the development and validation of the MuGIPS could contribute to a holistic study of gender inequalities by encompassing different domains in which gender inequality can be observed using respondents’ perceptions of inequality within the society and social circles in which they reside as a reference. The current research, with its potential impact on inequality reduction, could inspire future studies, especially given the link between perceived gender inequality and support for collective action. Forthcoming research could evaluate factors that promote perceptions of gender inequality as measured by the MuGIPS and other factors such as emotions [76] or attitudes toward empowerment [77], that may enhance the relationship between the MuGIPS and actions to reduce gender inequality in different settings. Thus, given the dramatic consequences of gender inequality [15–26], we believe that this instrument might be relevant to developing and improving intervention strategies to increase awareness of gender inequality and, especially, evaluate its effectiveness. Moreover, measuring various variables and selecting different outcomes related to gender inequality, as well as other psychosocial phenomena such as subjective wellbeing, status anxiety, or motivation to address gender and other inequalities, would help provide more information regarding the place of gender inequality perception, and therefore our instrument, in the nomological network [78]. It would also be necessary to explore the relationship between the levels of perceived inequality and levels of actual gender inequality. Future studies could explore the validation of the scale in English and other languages, as well as its adaptation to various cultural contexts, fostering cross-national and cross-cultural understanding of gender inequality perception.

In conclusion, the MuGIPS not only represents a significant step forward in understanding gender inequality perceptions, but also includes factors that have been largely overlooked (e.g., health) and have now been identified as sources of inequality. Additionally, the instrument has been validated in the Spanish context, offering a valuable contribution beyond the traditionally dominant Anglo-centric research landscape.

## Supporting information

S1 Table Characteristics and sociodemographic data of Samples 1–3. (PDF)

S2 Table Frequencies in item response for Samples 1 and 2. (PDF)

S3 Table Final version of the MuGIPS’ items in Spanish (original). (PDF)

S4 Table Adjustment indexes for models tested with Confirmatory Factor Analysis (CFA). (PDF)

S1 File Logistic regressions. (PDF)

S2 File Mean differences in MuGIPS by demographics. (PDF)
