# Supplementary material for: Development and validation of the Multidimensional Gender Inequality Perception Scale (MuGIPS)
Source: PLoS One. 2024 Apr 18;19(4):e0301755. doi: 10.1371/journal.pone.0301755 (PMC11025890; doi:10.1371/journal.pone.0301755)
Supplement: S1 Table — (PDF) [file pone.0301755.s001.pdf]

S1 Table 1. Characteristics and sociodemographic data of Samples 1-3

	**Sample 1 (N=673)**	**Sample 2 (N=498)**	**Sample 3 (N=558)**
Purpose	Exploratory FactorAnalysis	Confirmatory Factor Analysis	Discriminant Validity
Sample type	University Students	General Population	University students (84.1%) and Staff (16%)
Gender	57.8% women	53.2% women	67.9% women; 1.4% other; 1.8% I prefer not to answer
Age (M, SD)	23.29 (5.39) min=18; max=63	35.35 (13.23) min=18; max=71	24.9 (9.04) min=18; max=73
Country of residence (%)			
Spain	99%	98%	98.2%
Other	1%	2%	1.8%
Mother tongue (%)			
Spanish	94.2%	93.4%	97.3%
Other	5.8%	6.6%	2.7%
Sexual orientation (%)			
Heterosexual	70%	85.7%	66.7%
Homosexual	8%	2.2%	7.7%
Bisexual	18.6%	10.6%	20.3%
Other	1.3%	0.8%	1.4%
Prefer not to say	2.1%	0.6%	3.9%
Higher educational level achieved (%)			
No education	0%	0.2%	0%
Primary School	0%	1.6%	0.4%
Secondary school	0.3%	4.0%	0.2%
Vocational training	1.9%	12.2%	1.6%
High School diploma	7.6%	8.6%	11.3%
Bachelor’s degree	62.6%	38.8%	62.5%
Master’s degree	22.9%	28.3%	18.6%
PhD	4.8%	6.2%	5.4%
Occupation			

Full time employee	9.7%	43.8%	17.7%
Part time employee	5.3%	13.7%	7.5%
Freelancer	0.9%	6.0%	0.4%
Unemployed	5.5%	17.1%	5.7%
ERTE*	0.9%	2.8%	0%
Retired	0.1%	3.2%	0.7%
Student	76.7%	11.2%	74.2%
Work incapacity	0.1%	0.4%	0%
Other	0.7%	1.8%	1.1%
Annual income			
<10000€	9.5%	10.8%	11.3%
10000€ - 19999€	28.2%	24.1%	25.4%
20000€ - 29999€	19.3%	22.7%	24.2%
30000€ - 39999€	9.4%	14.7%	11.3%
40000€ - 49999€	9.2%	7.6%	6.8%
50000€ - 59999€	5.2%	6.2%	4.3%
60000€ - 69999€	2.5%	4.4%	3.6%
70000€ - 79999€	1.3%	2.2%	1.4%
80000€ - 89999€	1%	0.4%	0.4%
< 90000€	0.7%	1.8%	0.9%
I don’t know	12.3%	5%	10%
People living in the house (*M*, *SD*)	3.63 (1.29)	2.91 (1.13)	3.49 (1.27)
People underage living in the house (*M*, *SD*)	0.18 (0.56)	0.24 (0.61)	0.19 (0.65)
Political orientation (*M*, *SD*)	3.8 (1.66)	4.07 (1.65)	3.92 (1.79)
Subjective Social Status (*M*, *SD*)	5.14 (1.35)	5.13 (1.51)	5.34 (1.39)
MuGIPS Score (*M*, *SD*)	5.15 (1.10)	4.96 (1.11)	5.23 (1.19)
