# Supplementary material for: Development and validation of the Multidimensional Gender Inequality Perception Scale (MuGIPS)
Source: PLoS One. 2024 Apr 18;19(4):e0301755. doi: 10.1371/journal.pone.0301755 (PMC11025890; doi:10.1371/journal.pone.0301755)
Supplement: S2 Table — (PDF) [file pone.0301755.s002.pdf]

S2 Table. Frequencies in item response for Samples 1 and 2

**Item 1 -** Less attention is paid to the same health problem in women than in men.												
	**Combined Samples 1 & 2** total N=1171, valid N=1171, M=3.09, SD=1.96				**Sample 1** N=673, valid N=673, M=3.23, SD=2.01				**Sample 2** N=498, valid N=498, M=2.91,SD =1.87			
label	*frq*	*raw.prc*	*valid.prc*	*cum.prc*	*frq*	*raw.prc*	*valid.prc*	*cum.prc*	*frq*	*raw.prc*	*valid.prc*	*cum.prc*
1. Never (1)	246	21.01	21.01	21.01	128	19.02	19.02	19.02	118	23.69	23.69	23.69
2 (2)	403	34.42	34.42	55.42	227	33.73	33.73	52.75	176	35.34	35.34	59.04
3 (3)	82	7.00	7.00	62.43	45	6.69	6.69	59.44	37	7.43	7.43	66.47
4 (4)	169	14.43	14.43	76.86	96	14.26	14.26	73.70	73	14.66	14.66	81.12
5 (5)	118	10.08	10.08	86.93	81	12.04	12.04	85.74	37	7.43	7.43	88.55
6 (6)	67	5.72	5.72	92.66	43	6.39	6.39	92.12	24	4.82	4.82	93.37
7. Always (7)	32	2.73	2.73	95.39	14	2.08	2.08	94.21	18	3.61	3.61	96.99
I prefer not to answer (8)	54	4.61	4.61	100.00	39	5.79	5.79	100.00	15	3.01	3.01	100.00
NA	0	0.00	NA	NA	0	0.00	NA	NA	0	0.00	NA	NA

**Item 2 -** Women's specific health problems are underestimated compared to men's specific health problems.												
	**Combined Samples 1 & 2** total N=1171, valid N=1171, M=3.63, SD=2.03				**Sample 1** N=673, valid N=673, M=3.75, SD=2.05				**Sample 2** N=498, valid N=498, M=2.46, SD =1.98			
label	*frq*	*raw.prc*	*valid.prc*	*cum.prc*	*frq*	*raw.prc*	*valid.prc*	*cum.prc*	*frq*	*raw.prc*	*valid.prc*	*cum.prc*
1. Never (1)	168	14.35	14.35	14.35	93	13.82	13.82	13.82	75	15.06	15.06	15.06
2 (2)	320	27.33	27.33	41.67	167	24.81	24.81	38.63	153	30.72	30.72	45.78
3 (3)	98	8.37	8.37	50.04	61	9.06	9.06	47.70	37	7.43	7.43	53.21
4 (4)	191	16.31	16.31	66.35	103	15.30	15.30	63.00	88	17.67	17.67	70.88
5 (5)	160	13.66	13.66	80.02	102	15.16	15.16	78.16	58	11.65	11.65	82.53
6 (6)	104	8.88	8.88	88.90	66	9.81	9.81	87.96	38	7.63	7.63	90.16
7. Always (7)	83	7.09	7.09	95.99	50	7.43	7.43	95.39	33	6.63	6.63	96.79
I prefer not to answer (8)	47	4.01	4.01	100.00	31	4.61	4.61	100.00	16	3.21	3.21	100.00
NA	0	0.00	NA	NA	0	0.00	NA	NA	0	0.00	NA	NA

Deleted Item 1* - Care and hygiene products for women are more expensive than those for men (razors, shampoos, perfumes, creams, etc.).

	**Combined Samples 1 & 2** total N=1171, valid N=1171, M=5.54, SD=1.70				**Sample 1** N=673, valid N=673, M=5.70, SD=1.61				**Sample 2** N=498, valid N=498, M=5.33, SD =1.78			
Label (value)	*frq*	*raw.prc*	*valid.prc*	*cum.prc*	*frq*	*raw.prc*	*valid.prc*	*cum.prc*	*frq*	*raw.prc*	*valid.prc*	*cum.prc*
1. Never (1)	27	2.31	2.31	2.31	13	1.93	1.93	1.93	14	2.81	2.81	2.81
2 (2)	70	5.98	5.98	8.28	31	4.61	4.61	6.54	39	7.83	7.83	10.64
3 (3)	54	4.61	4.61	12.89	29	4.31	4.31	10.85	25	5.02	5.02	15.66
4 (4)	133	11.36	11.36	24.25	64	9.51	9.51	20.36	69	13.86	13.86	29.52
5 (5)	191	16.31	16.31	40.56	108	16.05	16.05	36.40	83	16.67	16.67	46.18
6 (6)	262	22.37	22.37	62.94	151	22.44	22.44	58.84	111	22.29	22.29	68.47
7. Always (7)	375	32.02	32.02	94.96	247	36.70	36.70	95.54	128	25.70	25.70	94.18
I prefer not to answer (8)	59	5.04	5.04	100.00	30	4.46	4.46	100.00	29	5.82	5.82	100.00
NA	0	0.00	NA	NA	0	0.00	NA	NA	0	0.00	NA	NA

**Item 3 -** Girls and boys are educated differently about their roles in society.												
	**Combined Samples 1 & 2** total N=1171, valid N=1171, M=4.72, SD=1.79				**Sample 1** N=673, valid N=673, M=4.84, SD=1.83				**Sample 2** N=498, valid N=498, M=4.56, SD =1.72			
Label (value)	*frq*	*raw.prc*	*valid.prc*	*cum.prc*	*frq*	*raw.prc*	*valid.prc*	*cum.prc*	*frq*	*raw.prc*	*valid.prc*	*cum.prc*
1. Never (1)	66	5.64	5.64	5.64	35	5.20	5.20	5.20	31	6.22	6.22	6.22
2 (2)	129	11.02	11.02	16.65	79	11.74	11.74	16.94	50	10.04	10.04	16.27
3 (3)	82	7.00	7.00	23.65	40	5.94	5.94	22.88	42	8.43	8.43	24.70
4 (4)	180	15.37	15.37	39.03	90	13.37	13.37	36.26	90	18.07	18.07	42.77
5 (5)	259	22.12	22.12	61.14	136	20.21	20.21	56.46	123	24.70	24.70	67.47
6 (6)	257	21.95	21.95	83.09	157	23.33	23.33	79.79	100	20.08	20.08	87.55
7. Always (7)	188	16.05	16.05	99.15	129	19.17	19.17	98.96	59	11.85	11.85	99.40
I prefer not to answer (8)	10	0.85	0.85	100.00	7	1.04	1.04	100.00	3	0.60	0.60	100.00
NA	0	0.00	NA	NA	0	0.00	NA	NA	0	0.00	NA	NA

**Item 4 -** University studies that are mainly taken by men are more valued than those taken by women.												
	**Combined Samples 1 & 2** total N=1171, valid N=1171, M=4.20, SD=1.90				**Sample 1** N=673, valid N=673, M=4.33, SD=1.93				**Sample 2** N=498, valid N=498, M=4.02, SD =1.83			
Label (value)	*frq*	*raw.prc*	*valid.prc*	*cum.prc*	*frq*	*raw.prc*	*valid.prc*	*cum.prc*	*frq*	*raw.prc*	*valid.prc*	*cum.prc*
1. Never (1)	113	9.65	9.65	9.65	61	9.06	9.06	9.06	52	10.44	10.44	10.44
2 (2)	185	15.80	15.80	25.45	106	15.75	15.75	24.81	79	15.86	15.86	26.31
3 (3)	98	8.37	8.37	33.82	49	7.28	7.28	32.10	49	9.84	9.84	36.14
4 (4)	224	19.13	19.13	52.95	113	16.79	16.79	48.89	111	22.29	22.29	58.43
5 (5)	225	19.21	19.21	72.16	129	19.17	19.17	68.05	96	19.28	19.28	77.71
6 (6)	189	16.14	16.14	88.30	126	18.72	18.72	86.78	63	12.65	12.65	90.36
7. Always (7)	110	9.39	9.39	97.69	70	10.40	10.40	97.18	40	8.03	8.03	98.39
I prefer not to answer (8)	27	2.31	2.31	100.00	19	2.82	2.82	100.00	8	1.61	1.61	100.00
NA	0	0.00	NA	NA	0	0.00	NA	NA	0	0.00	NA	NA

**Item 5 -** Women encounter more obstacles than men in pursuing their studies.												
	**Combined Samples 1 & 2** total N=1171, valid N=1171, M=3.67, SD=1.68				**Sample 1** N=673, valid N=673, M=3.77, SD=1.66				**Sample 2** N=498, valid N=498, M=3.54, SD =1.69			
Label (value)	*frq*	*raw.prc*	*valid.prc*	*cum.prc*	*frq*	*raw.prc*	*valid.prc*	*cum.prc*	*frq*	*raw.prc*	*valid.prc*	*cum.prc*
1. Never (1)	104	8.88	8.88	8.88	55	8.17	8.17	8.17	49	9.84	9.84	9.84
2 (2)	280	23.91	23.91	32.79	144	21.40	21.40	29.57	136	27.31	27.31	37.15
3 (3)	120	10.25	10.25	43.04	73	10.85	10.85	40.42	47	9.44	9.44	46.59
4 (4)	301	25.70	25.70	68.74	174	25.85	25.85	66.27	127	25.50	25.50	72.09
5 (5)	188	16.05	16.05	84.80	118	17.53	17.53	83.80	70	14.06	14.06	86.14
6 (6)	121	10.33	10.33	95.13	77	11.44	11.44	95.25	44	8.84	8.84	94.98
7. Always (7)	49	4.18	4.18	99.32	28	4.16	4.16	99.41	21	4.22	4.22	99.20
I prefer not to answer (8)	8	0.68	0.68	100.00	4	0.59	0.59	100.00	4	0.80	0.80	100.00
NA	0	0.00	NA	NA	0	0.00	NA	NA	0	0.00	NA	NA

**Item 6 -** Women experience more insecurity because of the possibility of being assaulted in everyday contexts (in the street, at work...) than men.												
	**Combined Samples 1 & 2** total N=1171, valid N=1171, M=6.11, SD=1.24				**Sample 1** N=673, valid N=673, M=6.26, SD=1.13				**Sample 2** N=498, valid N=498, M=5.91, SD =1.35			
Label (value)	*frq*	*raw.prc*	*valid.prc*	*cum.prc*	*frq*	*raw.prc*	*valid.prc*	*cum.prc*	*frq*	*raw.prc*	*valid.prc*	*cum.prc*
1. Never (1)	3	0.26	0.26	0.26	0	0.00	0.00	0.00	3	0.60	0.60	0.60
2 (2)	23	1.96	1.96	2.22	10	1.49	1.49	1.49	13	2.61	2.61	3.21
3 (3)	35	2.99	2.99	5.21	17	2.53	2.53	4.01	18	3.61	3.61	6.83
4 (4)	64	5.47	5.47	10.67	28	4.16	4.16	8.17	36	7.23	7.23	14.06
5 (5)	163	13.92	13.92	24.59	77	11.44	11.44	19.61	86	17.27	17.27	31.33
6 (6)	258	22.03	22.03	46.63	147	21.84	21.84	41.46	111	22.29	22.29	53.61
7. Always (7)	621	53.03	53.03	99.66	392	58.25	58.25	99.70	229	45.98	45.98	99.60
I prefer not to answer (8)	4	0.34	0.34	100.00	2	0.30	0.30	100.00	2	0.40	0.40	100.00
NA	0	0.00	NA	NA	0	0.00	NA	NA	0	0.00	NA	NA

**Item 7 -** Women suffer more from violence than men because they are women.												
	**Combined Samples 1 & 2** total N=1171, valid N=1171, M=5.70, SD=1.57				**Sample 1** N=673, valid N=673, M=5.79, SD=1.56				**Sample 2** N=498, valid N=498, M=5.58, SD =1.57			
Label (value)	*frq*	*raw.prc*	*valid.prc*	*cum.prc*	*frq*	*raw.prc*	*valid.prc*	*cum.prc*	*frq*	*raw.prc*	*valid.prc*	*cum.prc*
1. Never (1)	41	3.50	3.50	3.50	23	3.42	3.42	3.42	18	3.61	3.61	3.61
2 (2)	39	3.33	3.33	6.83	20	2.97	2.97	6.39	19	3.82	3.82	7.43
3 (3)	36	3.07	3.07	9.91	23	3.42	3.42	9.81	13	2.61	2.61	10.04
4 (4)	72	6.15	6.15	16.05	37	5.50	5.50	15.30	35	7.03	7.03	17.07
5 (5)	209	17.85	17.85	33.90	100	14.86	14.86	30.16	109	21.89	21.89	38.96
6 (6)	317	27.07	27.07	60.97	177	26.30	26.30	56.46	140	28.11	28.11	67.07
7. Always (7)	442	37.75	37.75	98.72	287	42.64	42.64	99.11	155	31.12	31.12	98.19
I prefer not to answer (8)	15	1.28	1.28	100.00	6	0.89	0.89	100.00	9	1.81	1.81	100.00
NA	0	0.00	NA	NA	0	0.00	NA	NA	0	0.00	NA	NA

**Item 8 -** Women are sexually assaulted to a greater extent than men.												
	**Combined Samples 1 & 2** total N=1171, valid N=1171, M=6.39, SD=0.93				**Sample 1** N=673, valid N=673, M=6.42, SD=0.92				**Sample 2** N=498, valid N=498, M=6.34, SD =0.96			
Label (value)	*frq*	*raw.prc*	*valid.prc*	*cum.prc*	*frq*	*raw.prc*	*valid.prc*	*cum.prc*	*frq*	*raw.prc*	*valid.prc*	*cum.prc*
1. Never (1)	3	0.26	0.26	0.26	2	0.30	0.30	0.30	1	0.20	0.20	0.20
2 (2)	5	0.43	0.43	0.68	3	0.45	0.45	0.74	2	0.40	0.40	0.60
3 (3)	12	1.02	1.02	1.71	6	0.89	0.89	1.63	6	1.20	1.20	1.81
4 (4)	32	2.73	2.73	4.44	16	2.38	2.38	4.01	16	3.21	3.21	5.02
5 (5)	102	8.71	8.71	13.15	53	7.88	7.88	11.89	49	9.84	9.84	14.86
6 (6)	335	28.61	28.61	41.76	189	28.08	28.08	39.97	146	29.32	29.32	44.18
7. Always (7)	675	57.64	57.64	99.40	401	59.58	59.58	99.55	274	55.02	55.02	99.20
I prefer not to answer (8)	7	0.60	0.60	100.00	3	0.45	0.45	100.00	4	0.80	0.80	100.00
NA	0	0.00	NA	NA	0	0.00	NA	NA	0	0.00	NA	NA

**Item 9 -** Women face more workplace harassment than men.												
	**Combined Samples 1 & 2** total N=1171, valid N=1171, M=5.69, SD=1.36				**Sample 1** N=673, valid N=673, M=5.82, SD=1.33				**Sample 2** N=498, valid N=498, M=5.51, SD =1.37			
Label (value)	*frq*	*raw.prc*	*valid.prc*	*cum.prc*	*frq*	*raw.prc*	*valid.prc*	*cum.prc*	*frq*	*raw.prc*	*valid.prc*	*cum.prc*
1. Never (1)	12	1.02	1.02	1.02	6	0.89	0.89	0.89	6	1.20	1.20	1.20
2 (2)	30	2.56	2.56	3.59	15	2.23	2.23	3.12	15	3.01	3.01	4.22
3 (3)	38	3.25	3.25	6.83	19	2.82	2.82	5.94	19	3.82	3.82	8.03
4 (4)	111	9.48	9.48	16.31	57	8.47	8.47	14.41	54	10.84	10.84	18.88
5 (5)	256	21.86	21.86	38.17	128	19.02	19.02	33.43	128	25.70	25.70	44.58
6 (6)	329	28.10	28.10	66.27	188	27.93	27.93	61.37	141	28.31	28.31	72.89
7. Always (7)	379	32.37	32.37	98.63	249	37.00	37.00	98.37	130	26.10	26.10	99.00
I prefer not to answer (8)	16	1.37	1.37	100.00	11	1.63	1.63	100.00	5	1.00	1.00	100.00
NA	0	0.00	NA	NA	0	0.00	NA	NA	0	0.00	NA	NA

**Item 10 -** Women are treated as objects to a greater extent than men.												
	**Combined Samples 1 & 2** total N=1171, valid N=1171, M=5.43, SD=1.48				**Sample 1** N=673, valid N=673, M=5.54, SD=1.42				**Sample 2** N=498, valid N=498, M=5.27, SD =1.54			
Label (value)	*frq*	*raw.prc*	*valid.prc*	*cum.prc*	*frq*	*raw.prc*	*valid.prc*	*cum.prc*	*frq*	*raw.prc*	*valid.prc*	*cum.prc*
1. Never (1)	24	2.05	2.05	2.05	11	1.63	1.63	1.63	13	2.61	2.61	2.61
2 (2)	49	4.18	4.18	6.23	25	3.71	3.71	5.35	24	4.82	4.82	7.43
3 (3)	54	4.61	4.61	10.85	23	3.42	3.42	8.77	31	6.22	6.22	13.65
4 (4)	113	9.65	9.65	20.50	64	9.51	9.51	18.28	49	9.84	9.84	23.49
5 (5)	289	24.68	24.68	45.18	159	23.63	23.63	41.90	130	26.10	26.10	49.60
6 (6)	325	27.75	27.75	72.93	192	28.53	28.53	70.43	133	26.71	26.71	76.31
7. Always (7)	312	26.64	26.64	99.57	196	29.12	29.12	99.55	116	23.29	23.29	99.60
I prefer not to answer (8)	5	0.43	0.43	100.00	3	0.45	0.45	100.00	2	0.40	0.40	100.00
NA	0	0.00	NA	NA	0	0.00	NA	NA	0	0.00	NA	NA

**Item 11 -** Women suffer more violence than men in intimate partner relationships.												
	**Combined Samples 1 & 2** total N=1171, valid N=1171, M=5.81, SD=1.21				**Sample 1** N=673, valid N=673, M=5.91, SD=1.16				**Sample 2** N=498, valid N=498, M=5.67, SD =1.27			
Label (value)	*frq*	*raw.prc*	*valid.prc*	*cum.prc*	*frq*	*raw.prc*	*valid.prc*	*cum.prc*	*frq*	*raw.prc*	*valid.prc*	*cum.prc*
1. Never (1)	17	1.45	1.45	1.45	10	1.49	1.49	1.49	7	1.41	1.41	1.41
2 (2)	10	0.85	0.85	2.31	4	0.59	0.59	2.08	6	1.20	1.20	2.61
3 (3)	32	2.73	2.73	5.04	14	2.08	2.08	4.16	18	3.61	3.61	6.22
4 (4)	81	6.92	6.92	11.96	34	5.05	5.05	9.21	47	9.44	9.44	15.66
5 (5)	196	16.74	16.74	28.69	104	15.45	15.45	24.67	92	18.47	18.47	34.14
6 (6)	490	41.84	41.84	70.54	292	43.39	43.39	68.05	198	39.76	39.76	73.90
7. Always (7)	335	28.61	28.61	99.15	209	31.05	31.05	99.11	126	25.30	25.30	99.20
I prefer not to answer (8)	10	0.85	0.85	100.00	6	0.89	0.89	100.00	4	0.80	0.80	100.00
NA	0	0.00	NA	NA	0	0.00	NA	NA	0	0.00	NA	NA

**Item 12 -** Women face more barriers to find employment than men.												
	**Combined Samples 1 & 2** total N=1171, valid N=1171, M=4.77, SD=1.53				**Sample 1** N=673, valid N=673, M=4.88, SD=1.54				**Sample 2** N=498, valid N=498, M=4.63, SD =1.51			
Label (value)	*frq*	*raw.prc*	*valid.prc*	*cum.prc*	*frq*	*raw.prc*	*valid.prc*	*cum.prc*	*frq*	*raw.prc*	*valid.prc*	*cum.prc*
1. Never (1)	38	3.25	3.25	3.25	22	3.27	3.27	3.27	16	3.21	3.21	3.21
2 (2)	79	6.75	6.75	9.99	39	5.79	5.79	9.06	40	8.03	8.03	11.24
3 (3)	98	8.37	8.37	18.36	56	8.32	8.32	17.38	42	8.43	8.43	19.68
4 (4)	232	19.81	19.81	38.17	117	17.38	17.38	34.77	115	23.09	23.09	42.77
5 (5)	317	27.07	27.07	65.24	187	27.79	27.79	62.56	130	26.10	26.10	68.88
6 (6)	267	22.80	22.80	88.04	156	23.18	23.18	85.74	111	22.29	22.29	91.16
7. Always (7)	134	11.44	11.44	99.49	92	13.67	13.67	99.41	42	8.43	8.43	99.60
I prefer not to answer (8)	6	0.51	0.51	100.00	4	0.59	0.59	100.00	2	0.40	0.40	100.00
NA	0	0.00	NA	NA	0	0.00	NA	NA	0	0.00	NA	NA

**Deleted Item 2* –** Unemployment rates are higher among women than among men.												
	**Combined Samples 1 & 2** total N=1171, valid N=1171, M=5.11, SD=1.58				**Sample 1** N=673, valid N=673, M=5.22, SD=1.55				**Sample 2** N=498, valid N=498, M=4.95, SD =1.61			
Label (value)	*frq*	*raw.prc*	*valid.prc*	*cum.prc*	*frq*	*raw.prc*	*valid.prc*	*cum.prc*	*frq*	*raw.prc*	*valid.prc*	*cum.prc*
1. Never (1)	20	1.71	1.71	1.71	13	1.93	1.93	1.93	7	1.41	1.41	1.41
2 (2)	50	4.27	4.27	5.98	33	4.90	4.90	6.84	17	3.41	3.41	4.82
3 (3)	52	4.44	4.44	10.42	31	4.61	4.61	11.44	21	4.22	4.22	9.04
4 (4)	192	16.40	16.40	26.81	125	18.57	18.57	30.01	67	13.45	13.45	22.49
5 (5)	304	25.96	25.96	52.78	170	25.26	25.26	55.27	134	26.91	26.91	49.40
6 (6)	210	17.93	17.93	70.71	120	17.83	17.83	73.11	90	18.07	18.07	67.47
7. Always (7)	199	16.99	16.99	87.70	100	14.86	14.86	87.96	99	19.88	19.88	87.35
I prefer not to answer (8)	144	12.30	12.30	100.00	81	12.04	12.04	100.00	63	12.65	12.65	100.00
NA	0	0.00	NA	NA	0	0.00	NA	NA	0	0.00	NA	NA

**Item 13 -** Women face more obstacles than men in accessing the most socially valued jobs.												
	**Combined Samples 1 & 2** total N=1171, valid N=1171, M=5.11, SD=1.58				**Sample 1** N=673, valid N=673, M=5.22, SD=1.55				**Sample 2** N=498, valid N=498, M=4.95, SD =1.61			
Label (value)	*frq*	*raw.prc*	*valid.prc*	*cum.prc*	*frq*	*raw.prc*	*valid.prc*	*cum.prc*	*frq*	*raw.prc*	*valid.prc*	*cum.prc*
1. Never (1)	34	2.90	2.90	2.90	17	2.53	2.53	2.53	17	3.41	3.41	3.41
2 (2)	76	6.49	6.49	9.39	38	5.65	5.65	8.17	38	7.63	7.63	11.04
3 (3)	70	5.98	5.98	15.37	38	5.65	5.65	13.82	32	6.43	6.43	17.47
4 (4)	156	13.32	13.32	28.69	78	11.59	11.59	25.41	78	15.66	15.66	33.13
5 (5)	285	24.34	24.34	53.03	166	24.67	24.67	50.07	119	23.90	23.90	57.03
6 (6)	327	27.92	27.92	80.96	197	29.27	29.27	79.35	130	26.10	26.10	83.13
7. Always (7)	211	18.02	18.02	98.98	128	19.02	19.02	98.37	83	16.67	16.67	99.80
I prefer not to answer (8)	12	1.02	1.02	100.00	11	1.63	1.63	100.00	1	0.20	0.20	100.00
NA	0	0.00	NA	NA	0	0.00	NA	NA	0	0.00	NA	NA

**Item 14 -** In our society there is a gender pay gap, i.e., men are paid more than women even though they do the same work.												
	**Combined Samples 1 & 2** total N=1171, valid N=1171, M=4.81, SD=1.69				**Sample 1** N=673, valid N=673, M=4.95, SD=1.64				**Sample 2** N=498, valid N=498, M=4.62, SD =1.74			
Label (value)	*frq*	*raw.prc*	*valid.prc*	*cum.prc*	*frq*	*raw.prc*	*valid.prc*	*cum.prc*	*frq*	*raw.prc*	*valid.prc*	*cum.prc*
1. Never (1)	69	5.89	5.89	5.89	36	5.35	5.35	5.35	33	6.63	6.63	6.63
2 (2)	83	7.09	7.09	12.98	35	5.20	5.20	10.55	48	9.64	9.64	16.27
3 (3)	79	6.75	6.75	19.73	42	6.24	6.24	16.79	37	7.43	7.43	23.69
4 (4)	178	15.20	15.20	34.93	95	14.12	14.12	30.91	83	16.67	16.67	40.36
5 (5)	315	26.90	26.90	61.83	195	28.97	28.97	59.88	120	24.10	24.10	64.46
6 (6)	278	23.74	23.74	85.57	163	24.22	24.22	84.10	115	23.09	23.09	87.55
7. Always (7)	150	12.81	12.81	98.38	93	13.82	13.82	97.92	57	11.45	11.45	99.00
I prefer not to answer (8)	19	1.62	1.62	100.00	14	2.08	2.08	100.00	5	1.00	1.00	100.00
NA	0	0.00	NA	NA	0	0.00	NA	NA	0	0.00	NA	NA

**Item 15 -** Men are more likely than women to have access to employment with better working conditions.												
	**Combined Samples 1 & 2** total N=1171, valid N=1171, M=4.68, SD=1.52				**Sample 1** N=673, valid N=673, M=4.74, SD=1.50				**Sample 2** N=498, valid N=498, M=4.60, SD =1.53			
Label (value)	*frq*	*raw.prc*	*valid.prc*	*cum.prc*	*frq*	*raw.prc*	*valid.prc*	*cum.prc*	*frq*	*raw.prc*	*valid.prc*	*cum.prc*
1. Never (1)	41	3.50	3.50	3.50	22	3.27	3.27	3.27	19	3.82	3.82	3.82
2 (2)	89	7.60	7.60	11.10	49	7.28	7.28	10.55	40	8.03	8.03	11.85
3 (3)	98	8.37	8.37	19.47	48	7.13	7.13	17.68	50	10.04	10.04	21.89
4 (4)	225	19.21	19.21	38.68	132	19.61	19.61	37.30	93	18.67	18.67	40.56
5 (5)	349	29.80	29.80	68.49	202	30.01	30.01	67.31	147	29.52	29.52	70.08
6 (6)	278	23.74	23.74	92.23	167	24.81	24.81	92.12	111	22.29	22.29	92.37
7. Always (7)	72	6.15	6.15	98.38	39	5.79	5.79	97.92	33	6.63	6.63	99.00
I prefer not to answer (8)	19	1.62	1.62	100.00	14	2.08	2.08	100.00	5	1.00	1.00	100.00
NA	0	0.00	NA	NA	0	0.00	NA	NA	0	0.00	NA	NA

**Deleted Item 3* -** Women that decide not to have children are questioned more than men who decide the same.												
	**Combined Samples 1 & 2** total N=1171, valid N=1171, M=5.72, SD=1.65				**Sample 1** N=673, valid N=673, M=5.84, SD=1.60				**Sample 2** N=498, valid N=498, M=5.56, SD =1.70			
Label (value)	*frq*	*raw.prc*	*valid.prc*	*cum.prc*	*frq*	*raw.prc*	*valid.prc*	*cum.prc*	*frq*	*raw.prc*	*valid.prc*	*cum.prc*
1. Never (1)	29	2.48	2.48	2.48	13	1.93	1.93	1.93	16	3.21	3.21	3.21
2 (2)	75	6.40	6.40	8.88	41	6.09	6.09	8.02	34	6.83	6.83	10.04
3 (3)	37	3.16	3.16	12.04	17	2.53	2.53	10.55	20	4.02	4.02	14.06
4 (4)	76	6.49	6.49	18.53	38	5.65	5.65	16.20	38	7.63	7.63	21.69
5 (5)	153	13.07	13.07	31.60	92	13.67	13.67	29.87	61	12.25	12.25	33.94
6 (6)	287	24.51	24.51	56.11	149	22.14	22.14	52.01	138	27.71	27.71	61.65
7. Always (7)	496	42.36	42.36	98.46	309	45.91	45.91	97.92	187	37.55	37.55	99.20
I prefer not to answer (8)	18	1.54	1.54	100.00	14	2.08	2.08	100.00	4	0.80	0.80	100.00
NA	0	0.00	NA	NA	0	0.00	NA	NA	0	0.00	NA	NA

**Item 16 -** Women take more responsibility for the caregiving of their children than men.												
	**Combined Samples 1 & 2** total N=1171, valid N=1171, M=5.47, SD=1.20				**Sample 1** N=673, valid N=673, M=5.48, SD=1.20				**Sample 2** N=498, valid N=498, M=5.47, SD =1.20			
Label (value)	*frq*	*raw.prc*	*valid.prc*	*cum.prc*	*frq*	*raw.prc*	*valid.prc*	*cum.prc*	*frq*	*raw.prc*	*valid.prc*	*cum.prc*
1. Never (1)	11	0.94	0.94	0.94	5	0.74	0.74	0.74	6	1.20	1.20	1.20
2 (2)	22	1.88	1.88	2.82	14	2.08	2.08	2.82	8	1.61	1.61	2.81
3 (3)	49	4.18	4.18	7.00	28	4.16	4.16	6.98	21	4.22	4.22	7.03
4 (4)	106	9.05	9.05	16.05	65	9.66	9.66	16.64	41	8.23	8.23	15.26
5 (5)	312	26.64	26.64	42.70	172	25.56	25.56	42.20	140	28.11	28.11	43.37
6 (6)	479	40.91	40.91	83.60	278	41.31	41.31	83.51	201	40.36	40.36	83.73
7. Always (7)	187	15.97	15.97	99.57	107	15.90	15.90	99.41	80	16.06	16.06	99.80
I prefer not to answer (8)	5	0.43	0.43	100.00	4	0.59	0.59	100.00	1	0.20	0.20	100.00
NA	0	0.00	NA	NA	0	0.00	NA	NA	0	0.00	NA	NA

**Item 17 -** Women do more housework than men.												
	**Combined Samples 1 & 2** total N=1171, valid N=1171, M=5.62, SD=1.14				**Sample 1** N=673, valid N=673, M=5.64, SD=1.12				**Sample 2** N=498, valid N=498, M=5.61, SD =1.18			
Label (value)	*frq*	*raw.prc*	*valid.prc*	*cum.prc*	*frq*	*raw.prc*	*valid.prc*	*cum.prc*	*frq*	*raw.prc*	*valid.prc*	*cum.prc*
1. Never (1)	8	0.68	0.68	0.68	3	0.45	0.45	0.45	5	1.00	1.00	1.00
2 (2)	16	1.37	1.37	2.05	11	1.63	1.63	2.08	5	1.00	1.00	2.01
3 (3)	32	2.73	2.73	4.78	15	2.23	2.23	4.31	17	3.41	3.41	5.42
4 (4)	93	7.94	7.94	12.72	48	7.13	7.13	11.44	45	9.04	9.04	14.46
5 (5)	307	26.22	26.22	38.94	189	28.08	28.08	39.52	118	23.69	23.69	38.15
6 (6)	472	40.31	40.31	79.25	270	40.12	40.12	79.64	202	40.56	40.56	78.71
7. Always (7)	233	19.90	19.90	99.15	129	19.17	19.17	98.81	104	20.88	20.88	99.60
I prefer not to answer (8)	10	0.85	0.85	100.00	8	1.19	1.19	100.00	2	0.40	0.40	100.00
NA	0	0.00	NA	NA	0	0.00	NA	NA	0	0.00	NA	NA

**Item 18 -** Women are more likely than men to engage in the caregiving for family members and others close to them.												
	**Combined Samples 1 & 2** total N=1171, valid N=1171, M=5.71, SD=1.09				**Sample 1** N=673, valid N=673, M=5.71, SD=1.09				**Sample 2** N=498, valid N=498, M=5.71, SD =1.08			
Label (value)	*frq*	*raw.prc*	*valid.prc*	*cum.prc*	*frq*	*raw.prc*	*valid.prc*	*cum.prc*	*frq*	*raw.prc*	*valid.prc*	*cum.prc*
1. Never (1)	3	0.26	0.26	0.26	1	0.15	0.15	0.15	2	0.40	0.40	0.40
2 (2)	14	1.20	1.20	1.45	11	1.63	1.63	1.78	3	0.60	0.60	1.00
3 (3)	39	3.33	3.33	4.78	19	2.82	2.82	4.61	20	4.02	4.02	5.02
4 (4)	83	7.09	7.09	11.87	49	7.28	7.28	11.89	34	6.83	6.83	11.85
5 (5)	235	20.07	20.07	31.94	136	20.21	20.21	32.10	99	19.88	19.88	31.73
6 (6)	549	46.88	46.88	78.82	314	46.66	46.66	78.75	235	47.19	47.19	78.92
7. Always (7)	244	20.84	20.84	99.66	141	20.95	20.95	99.70	103	20.68	20.68	99.60
I prefer not to answer (8)	4	0.34	0.34	100.00	2	0.30	0.30	100.00	2	0.40	0.40	100.00
NA	0	0.00	NA	NA	0	0.00	NA	NA	0	0.00	NA	NA

**Deleted Item 4* -** Men contribute more to household financial support than women do.												
	**Combined Samples 1 & 2** total N=1171, valid N=1171, M=4.12, SD=1.30				**Sample 1** N=673, valid N=673, M=4.12, SD=1.28				**Sample 2** N=498, valid N=498, M=4.11, SD =1.33			
Label (value)	*frq*	*raw.prc*	*valid.prc*	*cum.prc*	*frq*	*raw.prc*	*valid.prc*	*cum.prc*	*frq*	*raw.prc*	*valid.prc*	*cum.prc*
1. Never (1)	25	2.13	2.13	2.13	14	2.08	2.08	2.08	11	2.21	2.21	2.21
2 (2)	117	9.99	9.99	12.13	62	9.21	9.21	11.29	55	11.04	11.04	13.25
3 (3)	170	14.52	14.52	26.64	92	13.67	13.67	24.96	78	15.66	15.66	28.92
4 (4)	418	35.70	35.70	62.34	263	39.08	39.08	64.04	155	31.12	31.12	60.04
5 (5)	322	27.50	27.50	89.84	176	26.15	26.15	90.19	146	29.32	29.32	89.36
6 (6)	86	7.34	7.34	97.18	47	6.98	6.98	97.18	39	7.83	7.83	97.19
7. Always (7)	9	0.77	0.77	97.95	4	0.59	0.59	97.77	5	1.00	1.00	98.19
I prefer not to answer (8)	24	2.05	2.05	100.00	15	2.23	2.23	100.00	9	1.81	1.81	100.00
NA	0	0.00	NA	NA	0	0.00	NA	NA	0	0.00	NA	NA

**Item 19 -** The work-life balance is more difficult for women than for men.												
	**Combined Samples 1 & 2** total N=1171, valid N=1171, M=5.34, SD=1.58				**Sample 1** N=673, valid N=673, M=5.33, SD=1.58				**Sample 2** N=498, valid N=498, M=5.35, SD =1.57			
Label (value)	frq	raw.prc	valid.prc	cum.prc	frq	raw.prc	valid.prc	cum.prc	*frq*	*raw.prc*	*valid.prc*	*cum.prc*
1. Never (1)	29	2.48	2.48	2.48	15	2.23	2.23	2.23	14	2.81	2.81	2.81
2 (2)	55	4.70	4.70	7.17	34	5.05	5.05	7.28	21	4.22	4.22	7.03
3 (3)	69	5.89	5.89	13.07	41	6.09	6.09	13.37	28	5.62	5.62	12.65
4 (4)	142	12.13	12.13	25.19	82	12.18	12.18	25.56	60	12.05	12.05	24.70
5 (5)	247	21.09	21.09	46.29	140	20.80	20.80	46.36	107	21.49	21.49	46.18
6 (6)	315	26.90	26.90	73.19	185	27.49	27.49	73.85	130	26.10	26.10	72.29
7. Always (7)	295	25.19	25.19	98.38	162	24.07	24.07	97.92	133	26.71	26.71	99.00
I prefer not to answer (8)	19	1.62	1.62	100.00	14	2.08	2.08	100.00	5	1.00	1.00	100.00
NA	0	0.00	NA	NA	0	0.00	NA	NA	0	0.00	NA	NA

**Item 20 -** Women are under more pressure than men to give up their careers to take care of their families.												
	**Combined Samples 1 & 2** total N=1171, valid N=1171, M=5.06, SD=1.55				**Sample 1** N=673, valid N=673, M=5.07, SD=1.57				**Sample 2** N=498, valid N=498, M=5.05, SD =1.52			
Label (value)	*frq*	*raw.prc*	*valid.prc*	*cum.prc*	*frq*	*raw.prc*	*valid.prc*	*cum.prc*	*frq*	*raw.prc*	*valid.prc*	*cum.prc*
1. Never (1)	25	2.13	2.13	2.13	13	1.93	1.93	1.93	12	2.41	2.41	2.41
2 (2)	79	6.75	6.75	8.88	47	6.98	6.98	8.92	32	6.43	6.43	8.84
3 (3)	81	6.92	6.92	15.80	50	7.43	7.43	16.34	31	6.22	6.22	15.06
4 (4)	167	14.26	14.26	30.06	102	15.16	15.16	31.50	65	13.05	13.05	28.11
5 (5)	311	26.56	26.56	56.62	158	23.48	23.48	54.98	153	30.72	30.72	58.84
6 (6)	287	24.51	24.51	81.13	172	25.56	25.56	80.53	115	23.09	23.09	81.93
7. Always (7)	214	18.27	18.27	99.40	126	18.72	18.72	99.26	88	17.67	17.67	99.60
I prefer not to answer (8)	7	0.60	0.60	100.00	5	0.74	0.74	100.00	2	0.40	0.40	100.00
NA	0	0.00	NA	NA	0	0.00	NA	NA	0	0.00	NA	NA

**Item 21 -** Men have greater representation and power than women in private and public institutions.												
	**Combined Samples 1 & 2** total N=1171, valid N=1171, M=5.48, SD=1.43				**Sample 1** N=673, valid N=673, M=5.54, SD=1.44				**Sample 2** N=498, valid N=498, M=5.40, SD =1.42			
Label (value)	*frq*	*raw.prc*	*valid.prc*	*cum.prc*	*frq*	*raw.prc*	*valid.prc*	*cum.prc*	*frq*	*raw.prc*	*valid.prc*	*cum.prc*
1. Never (1)	21	1.79	1.79	1.79	13	1.93	1.93	1.93	8	1.61	1.61	1.61
2 (2)	42	3.59	3.59	5.38	21	3.12	3.12	5.05	21	4.22	4.22	5.82
3 (3)	58	4.95	4.95	10.33	34	5.05	5.05	10.10	24	4.82	4.82	10.64
4 (4)	97	8.28	8.28	18.62	48	7.13	7.13	17.24	49	9.84	9.84	20.48
5 (5)	279	23.83	23.83	42.44	159	23.63	23.63	40.86	120	24.10	24.10	44.58
6 (6)	370	31.60	31.60	74.04	207	30.76	30.76	71.62	163	32.73	32.73	77.31
7. Always (7)	294	25.11	25.11	99.15	183	27.19	27.19	98.81	111	22.29	22.29	99.60
I prefer not to answer (8)	10	0.85	0.85	100.00	8	1.19	1.19	100.00	2	0.40	0.40	100.00
NA	0	0.00	NA	NA	0	0.00	NA	NA	0	0.00	NA	NA

**Item 22 -** Although they have the same rights, men are socially respected more than women.												
	**Combined Samples 1 & 2** total N=1171, valid N=1171, M=4.87, SD=1.68				**Sample 1** N=673, valid N=673, M=5.03, SD=1.67				**Sample 2** N=498, valid N=498, M=4.65, SD =1.68			
Label (value)	*frq*	*raw.prc*	*valid.prc*	*cum.prc*	*frq*	*raw.prc*	*valid.prc*	*cum.prc*	*frq*	*raw.prc*	*valid.prc*	*cum.prc*
1. Never (1)	58	4.95	4.95	4.95	30	4.46	4.46	4.46	28	5.62	5.62	5.62
2 (2)	89	7.60	7.60	12.55	43	6.39	6.39	10.85	46	9.24	9.24	14.86
3 (3)	93	7.94	7.94	20.50	53	7.88	7.88	18.72	40	8.03	8.03	22.89
4 (4)	150	12.81	12.81	33.30	74	11.00	11.00	29.72	76	15.26	15.26	38.15
5 (5)	293	25.02	25.02	58.33	157	23.33	23.33	53.05	136	27.31	27.31	65.46
6 (6)	299	25.53	25.53	83.86	185	27.49	27.49	80.53	114	22.89	22.89	88.35
7. Always (7)	182	15.54	15.54	99.40	128	19.02	19.02	99.55	54	10.84	10.84	99.20
I prefer not to answer (8)	7	0.60	0.60	100.00	3	0.45	0.45	100.00	4	0.80	0.80	100.00
NA	0	0.00	NA	NA	0	0.00	NA	NA	0	0.00	NA	NA

**Item 23 -** Women are questioned more than men when they do not do what is expected of them.												
	**Combined Samples 1 & 2** total N=1171, valid N=1171, M=5.14, SD=1.74				**Sample 1** N=673, valid N=673, M=5.28, SD=1.72				**Sample 2** N=498, valid N=498, M=4.96, SD =1.76			
Label (value)	*frq*	*raw.prc*	*valid.prc*	*cum.prc*	*frq*	*raw.prc*	*valid.prc*	*cum.prc*	*frq*	*raw.prc*	*valid.prc*	*cum.prc*
1. Never (1)	48	4.10	4.10	4.10	25	3.71	3.71	3.71	23	4.62	4.62	4.62
2 (2)	98	8.37	8.37	12.47	48	7.13	7.13	10.85	50	10.04	10.04	14.66
3 (3)	58	4.95	4.95	17.42	28	4.16	4.16	15.01	30	6.02	6.02	20.68
4 (4)	128	10.93	10.93	28.35	80	11.89	11.89	26.89	48	9.64	9.64	30.32
5 (5)	254	21.69	21.69	50.04	133	19.76	19.76	46.66	121	24.30	24.30	54.62
6 (6)	291	24.85	24.85	74.89	161	23.92	23.92	70.58	130	26.10	26.10	80.72
7. Always (7)	276	23.57	23.57	98.46	187	27.79	27.79	98.37	89	17.87	17.87	98.59
I prefer not to answer (8)	18	1.54	1.54	100.00	11	1.63	1.63	100.00	7	1.41	1.41	100.00
NA	0	0.00	NA	NA	0	0.00	NA	NA	0	0.00	NA	NA

**Item 24 -** Men's opinions and ideas are more valued than those of women.												
	**Combined Samples 1 & 2** total N=1171, valid N=1171, M=4.56, SD=1.62				**Sample 1** N=673, valid N=673, M=4.63, SD=1.63				**Sample 2** N=498, valid N=498, M=4.47, SD=1.60			
Label (value)	*frq*	*raw.prc*	*valid.prc*	*cum.prc*	*frq*	*raw.prc*	*valid.prc*	*cum.prc*	*frq*	*raw.prc*	*valid.prc*	*cum.prc*
1. Never (1)	60	5.12	5.12	5.12	33	4.90	4.90	4.90	27	5.42	5.42	5.42
2 (2)	103	8.80	8.80	13.92	59	8.77	8.77	13.67	44	8.84	8.84	14.26
3 (3)	114	9.74	9.74	23.65	61	9.06	9.06	22.73	53	10.64	10.64	24.90
4 (4)	211	18.02	18.02	41.67	112	16.64	16.64	39.38	99	19.88	19.88	44.78
5 (5)	329	28.10	28.10	69.77	194	28.83	28.83	68.20	135	27.11	27.11	71.89
6 (6)	244	20.84	20.84	90.61	142	21.10	21.10	89.30	102	20.48	20.48	92.37
7. Always (7)	98	8.37	8.37	98.98	65	9.66	9.66	98.96	33	6.63	6.63	99.00
I prefer not to answer (8)	12	1.02	1.02	100.00	7	1.04	1.04	100.00	5	1.00	1.00	100.00
NA	0	0.00	NA	NA	0	0.00	NA	NA	0	0.00	NA	NA

**Item 25 -** In general, men have more power than women in our society.												
	**Combined Samples 1 & 2** total N=1171, valid N=1171, M=5.40, SD=1.48				**Sample 1** N=673, valid N=673, M=5.43, SD=1.53				**Sample 2** N=498, valid N=498, M=5.37, SD =1.41			
Label (value)	*frq*	*raw.prc*	*valid.prc*	*cum.prc*	*frq*	*raw.prc*	*valid.prc*	*cum.prc*	*frq*	*raw.prc*	*valid.prc*	*cum.prc*
1. Never (1)	26	2.22	2.22	2.22	17	2.53	2.53	2.53	9	1.81	1.81	1.81
2 (2)	54	4.61	4.61	6.83	35	5.20	5.20	7.73	19	3.82	3.82	5.62
3 (3)	52	4.44	4.44	11.27	30	4.46	4.46	12.18	22	4.42	4.42	10.04
4 (4)	103	8.80	8.80	20.07	47	6.98	6.98	19.17	56	11.24	11.24	21.29
5 (5)	281	24.00	24.00	44.06	158	23.48	23.48	42.64	123	24.70	24.70	45.98
6 (6)	371	31.68	31.68	75.75	208	30.91	30.91	73.55	163	32.73	32.73	78.71
7. Always (7)	276	23.57	23.57	99.32	173	25.71	25.71	99.26	103	20.68	20.68	99.40
I prefer not to answer (8)	8	0.68	0.68	100.00	5	0.74	0.74	100.00	3	0.60	0.60	100.00
NA	0	0.00	NA	NA	0	0.00	NA	NA	0	0.00	NA	NA

**Deleted Item 5 -** In our society, there is gender inequality between men and women.												
	**Combined Samples 1 & 2** total N=1171, valid N=1171, M=5.46, SD=1.58				**Sample 1** N=673, valid N=673, M=5.63, SD=1.50				**Sample 2** N=498, valid N=498, M=5.23, SD =165			
Label (value)	*frq*	*raw.prc*	*valid.prc*	*cum.prc*	*frq*	*raw.prc*	*valid.prc*	*cum.prc*	*frq*	*raw.prc*	*valid.prc*	*cum.prc*
1. Never (1)	28	2.39	2.39	2.39	10	1.49	1.49	1.49	18	3.61	3.61	3.61
2 (2)	47	4.01	4.01	6.40	24	3.57	3.57	5.05	23	4.62	4.62	8.23
3 (3)	62	5.29	5.29	11.70	27	4.01	4.01	9.06	35	7.03	7.03	15.26
4 (4)	135	11.53	11.53	23.23	73	10.85	10.85	19.91	62	12.45	12.45	27.71
5 (5)	266	22.72	22.72	45.94	148	21.99	21.99	41.90	118	23.69	23.69	51.41
6 (6)	227	19.39	19.39	65.33	124	18.42	18.42	60.33	103	20.68	20.68	72.09
7. Always (7)	397	33.90	33.90	99.23	263	39.08	39.08	99.41	134	26.91	26.91	99.00
I prefer not to answer (8)	9	0.77	0.77	100.00	4	0.59	0.59	100.00	5	1.00	1.00	100.00
NA	0	0.00	NA	NA	0	0.00	NA	NA	0	0.00	NA	NA

Note: *Deleted Item 1 was excluded due to its rate of response to the option "I prefer not to answer" across samples (>5%). Deleted Item 2 was excluded because of the same reason, moreover, participants indicated that they were aware of the existence of an official rate for unemployment, and they preferred not to answer because they didn't know the actual number of unemployed women and men. Deleted Item 3 was well captured by Item 23. Deleted Item 4 was excluded because its correlation with all the rest of the items were *rs* < .300. Deleted Item 5 was excluded for being too general.
