# Supplementary material for: Development and validation of the Multidimensional Gender Inequality Perception Scale (MuGIPS)
Source: PLoS One. 2024 Apr 18;19(4):e0301755. doi: 10.1371/journal.pone.0301755 (PMC11025890; doi:10.1371/journal.pone.0301755)
Supplement: S3 Table — (PDF) [file pone.0301755.s003.pdf]

S3 Table. Final version of the MuGIPS' items in Spanish (original).

Dimension	Item wording	#
Health	*Se presta menos atención a un mismo problema de salud cuando la paciente es mujer que cuando el paciente es hombre.*	1
	Algunos problemas de salud específicos de mujeres reciben menos atención que los problemas de salud específicos de hombres.	2
Violence	*Las mujeres experimentan más inseguridad por la posibilidad de ser agredidas en contextos cotidianos (en la calle, en el trabajo…) que los hombres.*	6
	Las mujeres, por el hecho de ser mujeres, son víctimas de más violencias que los hombres.	7
	Las mujeres son agredidas sexualmente en mayor medida que los hombres.	8
	Las mujeres sufren más acoso en el trabajo que los hombres.	9
	Las mujeres son tratadas como objetos en mayor medida que los hombres.	10
	Las mujeres sufren más violencia que los hombres en las relaciones de pareja.	11
Household work and caregiving	*Las mujeres se responsabilizan más del cuidado de sus hijas/os que los hombres.*	16
	Las mujeres realizan más tareas domésticas que los hombres.	17
	Las mujeres se encargan en mayor medida del cuidado de familiares y otras personas cercanas que los hombres.	18
Public sphere and power	(PR) *La conciliación de la vida personal y profesional es más difícil para las mujeres que para los hombres.*	19*
	(PR) *Las mujeres se ven más presionadas que los hombres para abandonar su carrera profesional y ocuparse de su familia.*	20*
	(PR) *Los hombres tienen mayor representación y poder que las mujeres en las instituciones tanto privadas como públicas.*	21
	(PR) *Aunque tienen los mismos derechos, socialmente se respeta más a los hombres que a las mujeres.*	22
	(PR) *Las mujeres son más cuestionadas que los hombres cuando no hacen lo que se espera de ellas.*	23

	(PR) *Las opiniones e ideas de los hombres se valoran más que las de las mujeres.*	24
	(PR) *En general, los hombres tienen más poder que las mujeres en nuestra sociedad.*	25
	(Edu) *Niñas y niños reciben una educación diferente sobre los roles que deben desempeñar en la sociedad.*	3
	(Edu) *Los estudios universitarios que cursan mayoritariamente los hombres se valoran más que los que cursan mayoritariamente las mujeres.*	4
	(Edu) *Las mujeres encuentran más obstáculos que los hombres para llevar a cabo sus estudios.*	5
	(EL) *Las mujeres se encuentran con más barreras que los hombres para conseguir un empleo.*	12
	(EL) *Las mujeres encuentran más obstáculos que los hombres para acceder a los puestos de trabajo mejor valorados socialmente.*	13
	(EL) *En nuestra sociedad existe una brecha salarial de género; es decir, aunque realicen el mismo trabajo, los hombres cobran más que las mujeres.*	14
	(EL) *Los hombres suelen acceder a un tipo de empleo con mejores condiciones laborales que las mujeres.*	15

Note: PR= theorized Power and Representation domain; Edu = theorized Education domain; EL = theorized Economics and Labor domain; * Items written to capture intersectional gender inequalities between the public sphere and representation dimension, and household work and caregiving dimension.
