# Supplementary material for: Development and validation of the Multidimensional Gender Inequality Perception Scale (MuGIPS)
Source: PLoS One. 2024 Apr 18;19(4):e0301755. doi: 10.1371/journal.pone.0301755 (PMC11025890; doi:10.1371/journal.pone.0301755)
Supplement: S4 Table — (PDF) [file pone.0301755.s004.pdf]

S4 Table. Adjustment indexes for models tested with Confirmatory Factor Analysis (CFA).

**Model**	**X^2(df)**	**CFI**	**TLI**	**RMSEA**	**SRMR**	**AIC**	**BIC**
1 factor	1266^{*} (230)	.816	.935	.099	.056	30108.1	30292.6
4 factor	621.1^{*}(224)	.947	.940	.06	.038	29472.2	29684.4
Second order with 4 factors	660.5^{*} (226)	.942	.847	.063	.043	29507.6	29711.7

Note: df = degrees of freedom; *p<.001
