# Supplementary material for: Development and validation of the Multidimensional Gender Inequality Perception Scale (MuGIPS)
Source: PLoS One. 2024 Apr 18;19(4):e0301755. doi: 10.1371/journal.pone.0301755 (PMC11025890; doi:10.1371/journal.pone.0301755)
Supplement: S1 File — (PDF) [file pone.0301755.s005.pdf]

S1 Analysis. Logistic regressions

We conducted logistic regressions to identify whether sociodemographic variables were associated with the likelihood of not responding to the items. We created a dummy variable (0 not responded, 1 responded), which was regressed on gender (1="man"; 2="woman"), age, political orientation (1="extreme left"; 10="extreme right"), subjective social class, and income level. We generally expected that men and older individuals would perceive less inequality and be more reluctant to respond to the proposed items.

We found that gender (B=-1.861, p<.001) and income level (B=.130; .004) were significant in predicting responses to the item "Care and hygiene products aimed at women are more expensive than those aimed at men (razors, shampoo, perfumes, creams...)"; this item was excluded given the fact that 5.1% participants selected the "I prefer not to answer" response. This indicates that men and people with higher incomes are more likely to avoid answering this item.

In the case of the item "Boys and girls receive a different education about the roles they should play in society" (0.8% of "I prefer not to answer" responses), gender was also a good predictor of the participants' response (B=-2.519; p=.017), with men being more likely to select the response "I prefer not to answer". Participants' gender also predited the "I prefer not to answer" response when the criterion variables were the items: "University studies mostly taken by men are valued more than those mostly taken by women"(B=-1.578; p=.001; 2.3% of "I prefer not to answer" responses); "Women, because they are women, are victims of more violence than men" (B=-1. 604; p=.014; 1.3% of responses "I prefer not to answer"); "Women suffer more violence than men in relationships"(B=-2.485; p=.019; 0. 8% of "I prefer not to answer" responses); "Men usually have access to a type of employment with better working conditions than women" (B=-1.392; p=.009; 1.6% of "I prefer not to answer"

responses); "Women are questioned more than men when they do not do what is expected of them" (B=-2.373, p=. 002; 1.5% of "I prefer not to answer" responses) and "Men's opinions and ideas are valued more than women's" (B=-1.841, p=.019; 1% of "I prefer not to answer" responses).

Gender and age also predicted the “I prefer not to answer” response, indicating that men and younger people are more likely to avoid answering the following items: "The unemployment rate is higher among women than among men" (B_{gender}=-.387, p_{gender}=.033; B_{age}= -.035, p_{age}=.001; this item was excluded because 12.4% of the participants selected "I prefer not to answer"); "Women have more obstacles than men in accessing the most socially valued jobs"(B_{gender}=-2.700, p_{gender}=.011; B_{age}=-.218, p_{age}=.024 1% of "I prefer not to answer" responses); "In our society there is a gender wage gap; that is, although they do the same work, men are paid more than women" (B_{gender}=-1.163, p_{gender}=.024; B_{age}=-.103, p_{age}=.048; 1. 6% of responses "I prefer not to answer"); "Women are questioned more than men when they decide not to have children" (B_{gender}=-1.904, p_{gender}=. 003; B_{age}=-.100, p_{age}=.045; 1. 6% of responses "I prefer not to answer") and "Work-life balance is more difficult for women than for men" (B_{gender}= -1.119, p_{gender}=.024; B_{age}=-.215, p_{age}=.006 1.6% of responses "I prefer not to answer").
