# Supplementary material for: Development and validation of the Multidimensional Gender Inequality Perception Scale (MuGIPS)
Source: PLoS One. 2024 Apr 18;19(4):e0301755. doi: 10.1371/journal.pone.0301755 (PMC11025890; doi:10.1371/journal.pone.0301755)
Supplement: S2 File — (PDF) [file pone.0301755.s006.pdf]

S2 Analysis. Mean differences in MuGIPS by demographics

In the case of gender, we compare men and women scores. Participants that indicated that they identify with other gender than men or women were less than 2% of the total sample, so we decided not to include them in this analysis.

Mean differences in MuGIPS scores by gender.

Variable	M (SD) by group	*t*
MuGIPS total score	Men=4.50(1.32)<br>Women=5.53 (0.99)	$t_{(236.69)} = -8.82$, $p < .001$, $d = .93$
Health	Men=2.88(1.66)<br>Women=3.72(1.69)	$t_{(537)} = -5.29$, $p < .001$, $d = .49$
Violence	Men=5.33(1.37)<br>Women=6.24 (0.90)	$t_{(218.91)} = -7.73$, $p < .001$, $d = .86$
Household Work and Caregiving	Men=5.14(1.26)<br>Women=5.85(1.01)	$t_{(248.97)} = -6.32$, $p < .001$, $d = .65$
Public Sphere and Power	Men=4.13(1.50)<br>Women=5.28(1.18)	$t_{(245.32)} = -8.58$, $p < .001$, $d = .89$

Regarding sexual orientation we considered heterosexual and bisexual respondents, since in both cases they were more than the 20% of the sample. Unfortunately, we didn't achieve large enough samples sizes to consider other sexual orientations.

Mean differences in MuGIPS scores by sexual orientation

Variable	M (SD) by group	*t*
MuGIPS total score	H=4.97(1.21)<br>B=5.90 (0.99)	$t_{(245.84)} = -8.77$, $p < .001$, $d = .84$
Health	H=3.11 (1.66)<br>B=4.51 (1.69)	$t_{(482)} = -7.96$, $p < .001$, $d = .83$
Violence	H=5.79 (1.22)<br>B=6.46 (0.74)	$t_{(308.25)} = -7.16$, $p < .001$, $d = .66$
Household Work and Caregiving	H=5.52(1.17)<br>B=5.93(1.08)	$t_{(198.89)} = -3.51$, $p < .001$, $d = .36$
Public Sphere and Power	H=4.64(1.38)<br>B=5.76(1.07)	$t_{(235.17)} = -9.05$, $p < .001$, $d = .90$

Note: group H = heterosexual, group B =bisexual

In the case of educational level, more than 60% of the sample had reached bachelor's level. None of the other educational levels concentrated the 20% or more of the sample, so we decided not to make comparisons between the levels of the educational level variable.

For the family annual income variable, we only considered cases of participants that selected as annual income “10000€ - 19999€ (2)” or “20000€ - 29999€ (3)”. The rest of the levels of the variable were selected by less than the 20% of the sample.

Mean differences in MuGIPS by income.

Variable	M (SD) by group	*t*
MuGIPS total score	2=5.52(1.06)<br>3=5.02 (1.34)	$t_{(255.54)} = 3.40$, $p < .001$,<br>$d = .41$
Health	2=3.81(1.64)<br>3=3.28(1.79)	$t_{(537)} = 2.58$, $p < .010$,<br>$d = .31$
Violence	2=6.24(0.89)<br>3=5.78(1.31)	$t_{(234.77)} = 3.40$, $p < .001$,<br>$d = .41$
Household Work and Caregiving	2=5.84(1.04)<br>3=5.51(1.28)	$t_{(248.76)} = 2.39$, $p = .017$,<br>$d = .28$
Public Sphere and Power	2=5.27(1.26)<br>3=4.72(1.55)	$t_{(258.79)} = 3.23$ $p = .001$,<br>$d = .40$

Note: group 2 = 10000€ - 19999€ per year; group 3 = 20000€ - 29999€ per year.

From these data, we can see that women perceive gender inequality to a greater extent than men. Bisexual people also perceive more gender inequality than heterosexual people. Lastly, people with lower incomes are more sensitised than people with somewhat higher annual incomes.
